# Significantly improved precision of cell migration analysis in time-lapse video microscopy through use of a fully automated tracking system

**DOI:** 10.1186/1471-2121-11-24

**Published:** 2010-04-08

**Authors:** Johannes Huth, Malte Buchholz, Johann M Kraus, Martin Schmucker, Götz von Wichert, Denis Krndija, Thomas Seufferlein, Thomas M Gress, Hans A Kestler

**Affiliations:** 1 Research group of Bioinformatics and Systems Biology, Institute of Neural Information Processing, Ulm University, Albert-Einstein-Allee 11, D-89081 Ulm, Germany; 2 Department of Gastroenterology and Endocrinology, University Hospital of Marburg, Germany; 3 Clinic of Internal Medicine I, Medical Centre Ulm University, Albert-Einstein-Allee 23, D-89081 Ulm, Germany; 4 Department of Internal Medicine I, Martin-Luther-University, Halle-Wittenberg, Germany

## Abstract

**Background:**

Cell motility is a critical parameter in many physiological as well as pathophysiological processes. In time-lapse video microscopy, manual cell tracking remains the most common method of analyzing migratory behavior of cell populations. In addition to being labor-intensive, this method is susceptible to user-dependent errors regarding the selection of "representative" subsets of cells and manual determination of precise cell positions.

**Results:**

We have quantitatively analyzed these error sources, demonstrating that manual cell tracking of pancreatic cancer cells lead to mis-calculation of migration rates of up to 410%. In order to provide for objective measurements of cell migration rates, we have employed multi-target tracking technologies commonly used in radar applications to develop fully automated cell identification and tracking system suitable for high throughput screening of video sequences of unstained living cells.

**Conclusion:**

We demonstrate that our automatic multi target tracking system identifies cell objects, follows individual cells and computes migration rates with high precision, clearly outperforming manual procedures.

## Background

The ability of individual cells to actively migrate, either randomly or directionally, across solid surfaces is an important biological parameter in many different contexts. During normal development, positioning of newly generated neurons through active migration is vital for the formation of a functional central and peripheral nervous system [1,2]. In the developed organism, cell motility is critical in processes such as wound healing, which requires fibroblasts and keratinocytes to migrate into wound sites [3,4], or the immune response, which involves extensive migratory activity of immune cells to and from lymphoid tissues and distant sites of infection [5-8]. In addition to these physiological roles, cell migration is also an important parameter in pathological processes such as carcinogenesis. Indeed, the acquisition of a distinct migratory potential is considered one of the hallmark features of malignant transformation of epithelial cells [9,10]. The molecular basis of tumor cell migration and its contribution to tumor progression, invasion and metastasis is thus an area of intense research [11-14].

A powerful method to directly observe and characterize the migratory behavior of cells is through the use of time-lapse microscopy [15-17]. Living cells are placed in appropriate culture media under a microscope and images of regions of interest are taken in regular intervals over extended periods of time. The positions of individual cells are then marked in consecutive images, thus following (tracking) positional changes of the cells over time. To date, this tracking procedure is commonly performed manually through "point and click" systems [5,11,12,18,19]. In addition to being labor-intensive, this method is highly susceptible to user-dependent errors regarding both the selection of "representative" subsets of cells for analysis (since rarely all cells in a given video sequence are considered) as well as the manual determination of cell centroids, which serve as measuring points for cell positions. In the current study, we have for the first time objectively quantified the magnitude of these error sources in manual cell tracking. Using migration of different populations of pancreatic cancer cells as a model system, we show that the results of manual cell tracking are highly variable and lead to mis-calculation of migration rates by up to 410%.

In order to avoid these error sources and provide objective measurements of cell migration rates, we have employed multi-target tracking technologies commonly used in military radar tracking applications [20,21] to develop a fully automated cell identification and tracking system suitable for high throughput screening of video sequences of unstained living cells. Image preprocessing and segmentation are adjusted to the high variability of microscopy image qualities, different cell sizes, cell shapes and general cell appearance. Tracking is performed on the sets of extracted cell centroids using a Kalman Filter implementation. Higher-level events, such as cell divisions or migration of cells out of and into the field of view, are automatically recognized and integrated into the analysis. We demonstrate that the system, which has been implemented as open source, cross-platform software, produces objective and highly reproducible measurements, clearly outperforming manual tracking procedures.

## Implementation and Methods

### Data and image sequence acquisition

The dataset consists of five unstained Panc1 cell image sequences (video samples). The cells were routinely kept in DMEM medium supplemented with 10% FCS in 5% CO_2 _atmosphere at 37°C. Before cytokine treatment, cells were kept in serum-free medium for 24 h. One sample was left untreated as a control group; the other cells were treated with substrates or substrate combinations including TGFb as a pro-migratory positive control.

The videos contain between 58 - 63 gray scale images (1024 × 1344 pixels, illumination intensity normalized between zero and one) and were recorded with a temporal resolution of t = 15 minutes and a magnification factor of 100. Each image pixel has a squared compass of 1.5 × 1.5 μm. Recording device was a Hamamatsu Orca camera. Acquisition technique was Differential Interference Contrast (DIC) microscopy.

Manual cell tracking was performed by experts for all cells that stayed within the region of analysis during the entire recording time (420 tracks) with ImageJ [22] using the *AviReader *Plugin (M. Schmid and D. Marsh) and the *Manual Tracking *plugin (F. Cordelieres) (see project website at http://rsbweb.nih.gov/ij/). All experiments were performed on an Intel Core 2 Duo, 2.4 GHz PC with 2 Gb RAM. Statistical analysis was performed with R http://www.r-project.org.

### Implementation

Our automatic tracking and analysis software was implemented using MatLab (v. 7.2) and consists of a graphical, cross platform open source application, adjustable to various types of microscopic images and video files. A modular architecture allows to expand image processing and tracking independently. The image processing unit provides miscellaneous image processing and segmentation functions freely combinable in a stack-like manner. For more complex configurations i.e. referencing previously processed images some additional functions are available. New MatLab image processing functions as well as program files written in C, C++ or Fortran, can be included into the program. We are constantly augmenting the functionality of the TimeLapseAnalyzer by adding new routines like wound healing assay analysis, cell counting, cell area measurements or image enhancement functions.

For more detailed information we refer to the Additional files 1 and 2.

## Results

### Variability of cell speed estimation caused by manual centroid selection

The migration rate of cells is commonly measured *via *the *mean displacement *(MD, i.e. the mean distance (μm) traveled per minute) of the cell centroid. The migratory potential of cell populations can then be expressed as the *average mean displacement *(AMD) of all cells in the analysis (see section "Cell migration rates" in the Additional File 1). In order to determine how the manual selection of the centroid positions of cells influences the calculated migration rates of individual cells and cell populations, we manually marked cells using a point-and-click system (see Materials and Methods) [22]. In a time-lapse video recording of Panc-1 pancreatic cancer cells, we tracked one cell repeatedly (40 times, one expert) across the sequence of 63 frames to obtain a realistic estimate of the variance in cell centroid selection introduced by manual cell tracking. For each frame, the mean value of the set of 40 clicked points was computed and subtracted from each point in the set. All sets were thereafter located around a zero mean and could be combined to a single set of 63 × 40 = 2520 points. To estimate the variance introduced by manual cell centroid selection, we assumed a single variability value for both the x and y coordinate (Ansari-Bradley test, p = 0.8199, 95% CI (confidence interval) for the ratio of the scales: 0.944 to 1.047, see e.g. Hajek and Sidak [23]), and pooled both x and y coordinate values to gain a single estimate for the standard deviation of the displacement values of ± 7.71 μm (5.14 pixels). Taking this as an upper variability value, we artificially imposed this type of centroid selection noise on three different manually selected tracks with low (sc 0.181 ± 0.213 μm/min), medium (mc 0.622 ± 0.411 μm/min) and high (fc 1.781 ± 0.821 μm/min) migration rates. The noise levels were 3, 4.5, 6, and 7.5 μm, corresponding to standard deviations of 2, 3, 4, and 5 pixels (see Table 1). We generated 200 tracks for each setting. Comparison of the AMD values of the noisy tracks with the MD of the original tracks revealed that the noisy tracks led to an overestimation of cell speeds averaging between 2% (fastest cell, 3 μm deviation) and 410% (slowest cell, 7.5 μm deviation) (see Table 1). Testing several filtering procedures for their ability to suppress the influence of the superimposed noise and to restore the original AMD measurements, we determined that smoothing of the noisy tracks prior to AMD calculation by a centered moving average filter (window size 5) produced AMD values that tended to slightly underestimate the original cell speeds, but were generally much closer to the "true" AMD values (Table 1).

**Table 1:** Cell speed variability caused by imprecise centroid selection

Cell type	σ in μm	original MD	AMD in μm/min	%	AMD (smoothed)	%
sc	3	0.181	0.423	234	0.125	69
sc	4.5		0.578	320	0.151	84
sc	6		0.747	414	0.181	100
sc	7.5		0.921	510	0.218	121

mc	3	0.622	0.723	116	0.465	75
mc	4.5		0.829	133	0.473	76
mc	6		0.956	154	0.484	78
mc	7.5		1.099	177	0.499	80

fc	3	1.787	1.821	102	1.589	89
fc	4.5		1.860	104	1.591	89
fc	6		1.923	108	1.597	89
fc	7.5		1.985	111	1.598	89

Three cell types where tested (slow cell (sc), medium fast cell (mc), fast cell (fc)). The centroid positions where artificially varied within a standard deviation (σ, both axes) of 3 to 7.5 μm around the real centroid and the average mean displacement (AMD) computed for each set of varied tracks (200 tracks per setting). Variation of centroid positions resulted in overestimation of cell speeds, which was most pronounced for the slowest cell. Smoothing of "noisy" cell tracks by a centered moving average filter (window size 5) tended to underestimate MD values to varying degrees.

### Variations induced by cell subset selection

**Figure 1:**
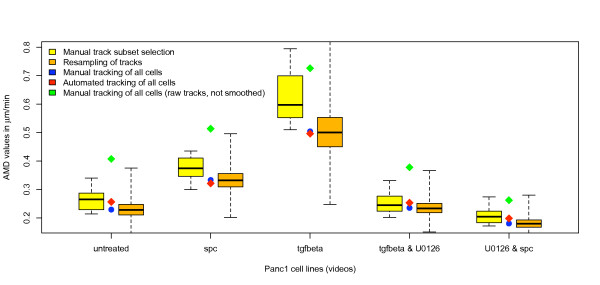
**Dependency of average mean displacement on track selection**. Variability of track set selection for average mean displacement calculation is shown for image sequences of five Panc1 cell lines treated with different compounds (spc: Sphingosylphosphorylcholine, TGFβ, U0126). All cells were tracked manually by one expert (overall track number n = 420; for cell numbers per video see Table 3). Ten subjects selected 20 of these tracks for average mean displacement calculation (yellow, boxplots showing median, interquartiles and range). Results of randomly sampling 20 of the tracks repeatedly for 2 × 10^5 ^times are shown as orange boxplots. Average mean displacement values, utilizing all available manually tracked cells are shown in blue (for raw not smoothed tracks in green). Results of automated tracking are given in red.

A common practice in manual cell tracking is to select only a subset of cells from each time-lapse video for analysis, which is then assumed to represent the whole cell population. We were interested in determining how closely manually selected subpopulations approximate the whole cell population in a typical experimental setting. To this end, we analyzed five video sequences of Panc-1 pancreatic cancer cells differentially treated with stimulatory and inhibitory substances. One expert manually tracked the cells in each of the five sample videos. Only tracks, which did not leave the field of view between the first and the last frame, were considered, resulting in a total of 420 valid tracks. AMD values were calculated for "raw" tracks as well as smoothened tracks (centered moving average filter, window size 5). The five video samples show well-distinguishable differences in migration rates regardless if "raw" or smoothened cell tracks were used for AMD computation (Figure 1). In order to isolate a possible bias resulting from subjective selection of cells from other error sources, we exclusively used smoothed tracks for AMD calculations, thereby excluding errors resulting from imprecise cell centroid selection as highlighted above. Ten participants were asked to choose a subset of 20 cells from each video, which they found to be good representatives of the cell population. The participants could observe the movement of the cells prior to their selection. They were however not informed about the treatment of the cells to avoid biasing the choice of cells due to *a priori *knowledge about expected effects of the inhibitory or stimulatory substances. The subsets chosen by each test person were used to compute the AMD for every video sample, revealing highly individual cell choices for the ten participants. In general, the selected subsets tended to substantially overestimate the average migration rates of the populations (Figure 1). The variance of the ten participants' choices was especially high for the samples with faster cells (i.e. the TGFβ- and SPC-treated cells; see Figure 1). To evaluate the degree of "agreement" between participants in selecting cells, we computed for any combination of two participants the number of commonly selected cells. The results demonstrate that across all 5 video sequences, on average 6.17 cells out of the 20 (max. 12; min. 1) were commonly chosen between any two participants (Table 2).

**Table 2:** Levels of agreement between any two participants in selecting "representative" subsets of 20 cells from cell populations.

Image sequence no.	Average number of common selected tracks for any 2 participants (out of 20 possible)
1, untreated	6.62 ± 2.29
2, spc	5.09 ± 1.90
3, tgfβ	7.82 ± 2.25
4, tgfβ & U0126	6.49 ± 2.05
5, U0126 & spc	4.84 ± 2.14

In order to estimate the total range of AMD values that can potentially result from selection of different 20-cell-subsets in each sample video, we performed repeated random sampling of 20 tracks (2 × 10^5 ^iterations per sample file). As shown in Figure 1 (orange boxplots), the range of possible values was extremely broad, reflecting a considerable range of migration rates among individual cells of a given population. Interestingly, the manually selected subset results were statistically significantly different from the random resampling results for four of the five sample files (Wilcoxon test with Bonferroni p-value adjustment: p_untreated _= 0.023, p_spc _= 0.011, p_tgfβ _= 0.00098, p_tgfβ & U0126 _= 0.893, p_U0126 & spc _= 0.0067), confirming that manual cell subset selection introduces significant bias in the data analysis.

### Automated cell tracking

In order to overcome the limitations of manual cell tracking, we have developed a fully automated image processing and tracking system comprising three stages: (a) cell centroid extraction on individual images, (b) tracking of individual cells centroids, and (c) track monitoring.

Identification of individual cells and extraction of geometrical cell centroids was performed by combining independent cell-background and cell-detail segmentation to maximize cell detection sensitivity and specificity.

**Figure 2:**
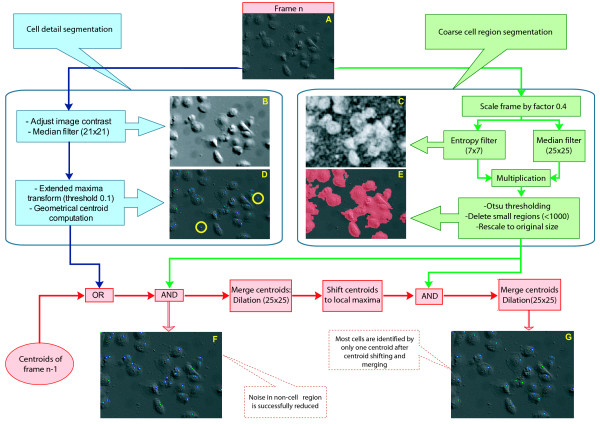
**Cell centroid segmentation**. Schematic workflow and examples of intermediary steps of cell centroid extraction from microscopic images. Each new frame (A) will be processed in two distinct steps, namely cell detail segmentation (left, blue box) and cell region segmentation (right, green box). The detected centroids from the detail segmentation are first combined with the extracted centroids of one past frame to propagate cell centroids steadily through an image sequence. Afterwards the combination of the cell region image and the cell centroid image leads to deletion of cell positions in non-cell regions (panel F). Subsequent centroid merging and shifting finally concentrate groups of possible centroids within one cell to form a single cell centroid (panel G).

For coarse cell region segmentation, we took advantage of structural discrepancies between cell tissue and image background. By computing the local image entropy, which measures the heterogeneity of intensity values in the neighborhood of each pixel, even strongly spread-out cells, which are most challenging to detect due to the low contrast they produce, are very efficiently detected. For detection of individual cell structures, we used local intensity thresholding, which is robust against illumination gradients across images. The subsequent combination of coarse cell region and cell detail images provides high cell detection sensitivity while noise in the media is successfully omitted. The entire image processing workflow is outlined in Figure 2.

As demonstrated in Table 3, the precision of automated cell identification and centroid placement was very high, resulting in cell detection rates ranging from 96 to 99%.

**Table 3:** Validity of automatically extracted cell tracks

Image sequence no.	Cell detection rate median (min, max)	# of frames/# of required cell-to-cell associations	Swap errors	Lost or deleted	Track detection (correct/total)	%
1, untreated	0.98 (0.92, 1.0)	63/4960	11	1	68/80	85
2, spc	0.98 (0.92, 1.0)	60/6077	9	4	90/103	87
3, tgfβ	0.96 (0.90, 0.99)	64/4284	17	2	49/68	73
4, tgfβ & U0126	0.97 (0.90, 1.0)	58/3933	3	3	63/69	91
5, U0126 & spc	0.99 (0.95, 1.0)	60/5900	6	5	89/100	89

Automatic track detection consists of cell identification and track generation. The cell identification rate is measured over all individual images. A cell track was counted as swapped and thus false in two cases: either if two tracks "exchanged" their cells (which leads to a double swapping error) or if the merging during the cell division (backward tracking) happened with the wrong child cell. The total number of cell-to-cell associations for each video file is given in column 3 (e.g., video 1 consists of 80 tracked cells over 63 frames, requiring a total of 62 × 80 = 4960 cell-to-cell associations. Only 11 of those were incorrect (0.2%), demonstrating an association performance of 99.8% for sample video 1).

The proportion of tracks that were followed correctly across all frames (i.e. without any form of mis-association of cells) is given in column 6. The third video clearly shows the highest swapping error, which was expected as it contains the fastest cells and the lowest cell detection sensitivity (0.96).

For the subsequent tracking of individual cell centroids through image sequences, Kalman filtering [24,25], commonly employed in multi-target tracking systems in military radar surveillance applications [20,21], was utilized. Kalman filters are a set of mathematical equations allowing "state ahead" predictions of object positions (cell centroids) as well as the estimation of optimized object states in noisy environments (e.g. resulting from variations in cell segmentation).

**Figure 3:**
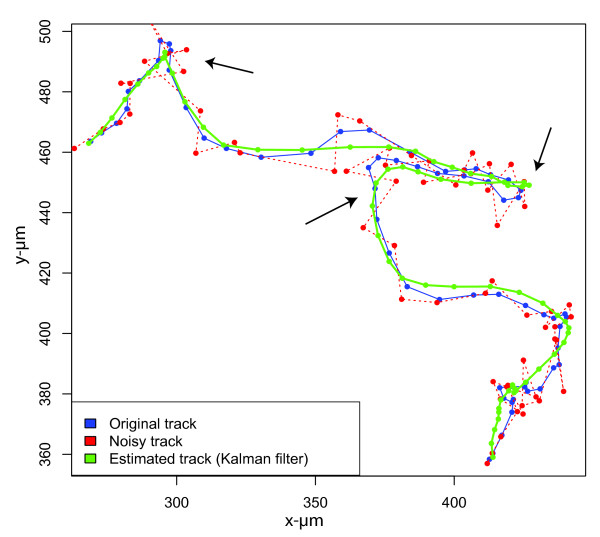
**Kalman filter tracked cell path**. The blue line displays the "ground truth" cell path without any influence of noise. The track was taken from the set of smoothed manual tracks of the first video file. The red dots indicate the noisy measurements, which were varied within a standard deviation of five pixels around the original (blue) path. The dashed red line shows the track that would result from taking the noisy measurements as real centroid positions. The track varies strongly around the original blue track. The green line displays the track derived by the Kalman Filter implemented in this project. A main part of the noise is successfully filtered with our approach so that the Kalman track appears much smoother than the track from the noisy measurement. Note that the KF with constant velocity model also performs well at major turning points of the trajectory (black arrows).

The applied discrete KF algorithm consists of two alternating steps, which are repeated in each iteration (for each new frame): prediction and correction. In the prediction step, the filter makes an assumption (*a priori*) about the future state of the observed object. In the correction step, an optimized (*a posteriori*) state estimate is computed using a *weighted *difference between the *a priori *state and an actual (noisy) measurement. The weighting term (K) is updated iteratively according to the quality of the previously *a priori *prediction: If the prediction was good, the weighting term will suppress the influence of the measurement in the next iteration and show more "trust" in the state ahead prediction than in the measurement. If the prediction was poor, K weights the measurement more heavily in the next iteration while suppressing the influence of the *a priori *estimate. An example of this "denoising" effect of Kalman filtering on cell tracks is shown in Figure 3. In our system, a constant velocity model was applied in the KF to predict future states of the objects. The model and the according variance were previously estimated using the manual extracted cell tracks (see Additional file 1).

To assign new measurements to each track end (i.e. measurement for the Kalman filter), the iterative unique nearest neighbor (UNN) algorithm was utilized. This algorithm associates only the best matching track-to-measurement pair in each loop and effectively guards against unreasonable track-to-measurement associations. The UNN algorithm terminates if either all tracks or all measurements are allocated.

In order to adequately analyze discontinuous cell tracks, we have implemented a Monitoring Module (MM), which recognizes and automatically integrates higher-level events, such as cell division or moving of cells into and out of the field of view, into the analysis. The likelihood of any such event is evaluated individually for each track based on the outcome of the UNN search, i.e. the distance between the previous track/new measurement pair. A first threshold determines if the pairing is likely to be correct. In this case the pair is accepted and no further actions are taken. If the distance of the pair is too high, possible alternatives are evaluated including cell division, track initialization, missing measurements, and movement of the cell out of the field of view. For each of these cases, an *event number *is defined which determines a maximal number of events before further actions are taken. For instance, if the measurement for a track is missing too often, the track terminates. Until this threshold is reached, the missing measurement is provided by the MM, i.e. it will be formed by the last determined cell position. Other higher level events are treated in a similar way, which effectively guards against segmentation and track-to-measurement association errors.

To simplify mitosis detection and track initialization/termination, we utilized backward tracking in our system, meaning that cells were followed from the last to the first frame [26]. In backward tracking, detection of cell division (mitosis) is observed as cell merging. This means that - during the course of a tracking analysis - cells can technically only newly emerge when they migrate into the field of view (thus only at the border of a frame) and false track initialization can effectively be avoided.

**Figure 4:**
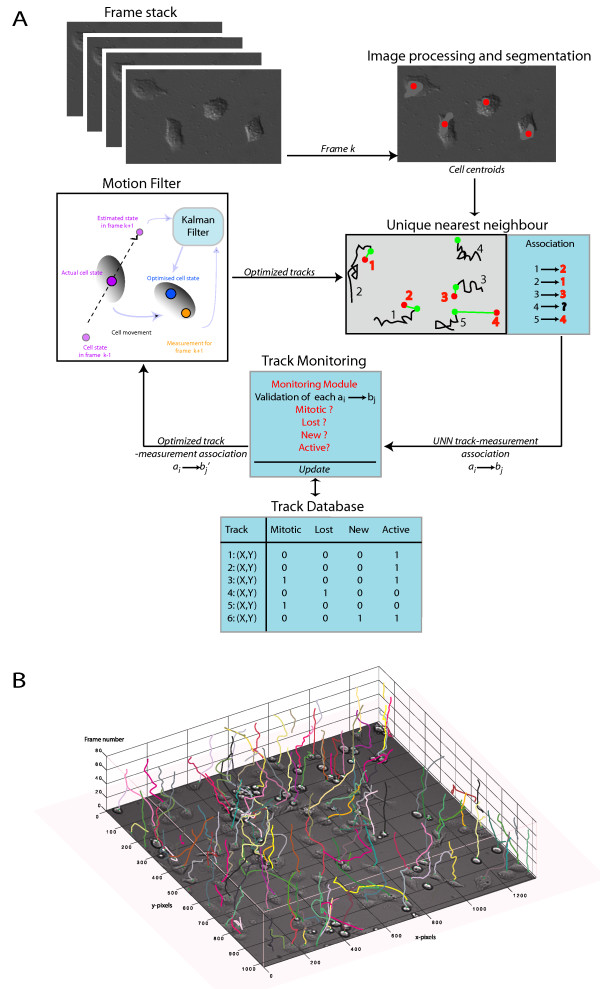
**Overview of the tracking scheme**. (A) In each iteration, the actual extracted cell centroids and the optimized state estimate from the Kalman filter process are used to compute the unique nearest neighbor for each track end. The unique nearest neighbor is processed in a monitoring module to check whether a cell division, cell death, or leaving of the cell out of view event might have occurred. The stored tracks are updated accordingly. All tracks that are still active are further processed: the tuple consisting of actual track end and associated unique nearest neighbor track (measurement) is used to make the next state ahead prediction using the Kalman filter. (B) Three-dimensional representation of the result of the migration analysis for a video sample derived by the automated tracking system. The extracted cell tracks are exemplarily plotted onto the first video frame. Each colored line marks the path of a single cell through the stack of images (video frames).

The complete automated tracking process, starting with the processed images, is schematically outlined in Figure 4A. The average computation time for one frame was five seconds. A detailed description of the cell segmentation, UNN, KF tracking and the MM as well as the user tunable parameters can be found in the supplementary material (Additional file 1). The entire system was implemented in MatLab as a graphical application (free, open source, cross platform). The image-processing module offers a large degree of adjustability to accommodate different cellular phenotypes (size, shape) or different image qualities (Additional file 2). The tracking module is adjustable to different cell speeds and types of motion. Examples of video files of Panc1 in DIC and HeLa cells recorded with phase contrast are part of the supplementary material (Additional files 3 to 5) accompanying this manuscript.

The software is available online (TimeLapseAnalyzer: Software Documentation: http://www.informatik.uni-ulm.de/ni/staff/HKestler/tla/) together with a short introduction, a detailed software documentation and example video files.

The tracking system was evaluated using the video sequences of differentially treated Panc-1 pancreatic cancer cells. Figure 4B provides a graphical representation of the tracking results for a sample video file. For evaluation of tracking performance, the complete set of 420 manually validated tracks (see Methods) was used to analyze the validity of corresponding automatically extracted tracks. An automatically generated track was only regarded as valid if it followed one cell (and only one) through all frames in which the cell was visible. This stringent criterion was violated if a track failed to initialize, was prematurely terminated, or swapped between two cells. The overall accuracy of the complete cell identification and tracking procedure across all five video samples was 85.48%. Swapping errors were highest for the fastest (TGFβ-treated) cells. In contrast, counts of lost or deleted cell tracks were uniformly low in all of the video files (Table 3). In order to evaluate the precision of cell speed measurements derived by the tracking system, AMD values calculated from automatically extracted tracks were compared to those calculated from smoothed manually determined tracks (centered moving average filter, window size 5). As shown above, the AMD values calculated from smoothed tracks provide the best possible estimate of "true" migration rates. No significant differences were detected between the automated tracking (Figure 1, red) and the manual tracking (Figure 1, blue) of all cells in the five image sequences (exact Wilcoxon test, paired, p = 0.25, 95% CI: -0.027 to 0.012 for difference in medians).

## Discussion and Conclusions

Active cell migration is a complex task involving many different cellular components and pathways. The identification and characterization of contributing factors is very important e.g. in cancer biology, where the migratory potential of malignant cells is directly related to their invasive and metastatic phenotype, and hence to patient prognosis. In order to be able to objectively evaluate the contribution of individual genes and specific signaling pathways, or to examine the influence of chemical compounds, etc., it is of utmost importance to measure migratory activity as precisely as possible.

### Error sources in manual cell tracking

In unstained cell images, cell borders can be difficult to detect visually. Together with the inherent difficulty of visually estimating the center of irregularly shaped objects, this leads to substantial imprecision in cell centroid determination in point-and-click methods of cell tracking. Bahnson et al. [27] have reported that manually determined cell centroid positions differed considerably between two individual analysis runs. In a study with synthetic data simulating the movement of fluorescent particles within cells, Smal et al. [28] estimated that the error of manual particle localization, even under these comparatively favorable conditions, was 2-3 times higher than the error of the automated tracking system they evaluated. Our own results with the real-world data sets revealed that the standard deviation of manually selected cell centroids from the estimated "true" cell centroids was as high as 7.71 μm for pancreatic cells, which display cell diameters of approx. 50 - 200 μm. As we have demonstrated, this consistently leads to overestimation of cell speeds by up to 410%.

Even more severe was the influence of cell subset selection on the result of the migration analyses. As mentioned above, selecting subsets of cells for analysis to approximate the behavior of the whole population is a common practice in manual cell tracking. The selection of cells from a video was found to be highly individual. Nearly all participants chose subsets that overrated the "true" migration rates. More importantly, variability of the results was precariously high between individual participants. This is also highlighted by the low level of agreement between the participants' cell subset choices (on the average only 6.17 out of 20 cells were mutually selected by any two participants). Although the relative differences of the AMD values between the single video files were preserved in all data sets for individual participants, these results clearly demonstrate that substantial user bias can be introduced in such an analysis which may be even more significant if certain experimental outcomes are expected *a-priori*. These uncertainties severely complicate the meaningful comparison of experimental results across different laboratories or different experimenters.

An obvious solution to the problem of human influence and subjective choices would be the random selection of cells without prior knowledge of the cells' behavior in the image sequence. Our resampling experiments, however, revealed that the range of possible outcomes using this selection strategy is extremely broad, posing a considerable danger of severely distorting the results of the analysis. Taken together, these results clearly demonstrate that manual tracking of cells, in particular when subsets of cells are used to approximate the behavior of whole populations, is not an adequate method to generate precise and inter-subjectively comparable measurements of cell motility. To our knowledge, this is the first report quantifying the influence of different error sources in manual cell tracking.

### Performance of the automated cell tracking system

Object identification is the first critical step in automated tracking applications [27,29]. The Differential Interference Contrast (DIC) imaging technique employed here offers the best prospect for recognition of unstained cells in live cell microscopy since it does not suffer from the phase halos typical of phase-contrast images [30]. However, precise identification of individual cells remains a challenging task for computer vision applications [31]. DIC images show no contrast perpendicular to the shear angle of the splitted beams, which excludes the use of the image processing techniques of skeletonization and standard contour closure to define the borders of cellular structures [32]. The low contrast regions which are typically encountered in strongly outspread migrating epithelial cells pose particular problems for the cell segmentation [33,34]. Previously proposed methods for cell identification in DIC images include template matching [35], local variance detection [36], or a combination of gradient variations and texture filter to outline cell boundaries Most recently, the use of deformable templates has been explored [27,37,38]. These are modeled closed curves, which are fitted to object boundaries in iterative processes. In each frame, an attraction area must be identified in the surrounding of each cell, either by seeking cell edges (gradients) which is less promising due to the missing contrast perpendicular to the shear angle, or by analyzing region-based energy [39,40]. However, all of these techniques are either limited to cell types with relatively constant sizes and shapes, or require relatively long processing times, making them unsuitable for high-throughput applications. We have demonstrated that the combined analysis of local image entropy [41] and local illumination intensity is suitable to identify individual cells with high sensitivity and specificity at low computational cost.

The precision of cell detection in our analyses ranged between 96% and 99%, which compares favorably with other systems, although only few related studies provide quantitative information regarding the performance of their cell identification procedures. For the segmentation of cells in a set of phase contrast videos, Li et al. [37] implemented a procedure of classifying pixels into foreground and background based on a coarse pre-segmentation and a maximum *a-posteriori *principle. They report a specificity of 98.1% and a sensitivity of 96.6% for the detection of individual cells. For the identification of fluorescent objects in live cell videos, several authors have used the well-established technique of watershed segmentation [42]. Chen et al. [43] and Yan et al. [44] report accuracies of detection of 97.8% and 98.12%, respectively, but had to implement fragment merging techniques to avoid over-segmentation, which is an inherent problem of the watershed segmentation principle.

The next step in our procedure is the tracking of individual detected cell centroids through the image sequences. The two main potential error sources during this phase are swapping of cells and erroneous loss or deletion of valid tracks. Of these, swapping of cells is less critical for the average mean displacement computation, since only single displacement values of individual tracks will be computed erroneously. In contrast, the deletion of tracks due to missing cell-to-cell associations can lead to larger errors in this calculation, as all displacement values beyond the time point where the track is deleted are lost for this measurement. We have implemented two procedures to guard against both types of errors: the Kalman Filter [24,25] and a Monitoring Module. Due to its computational simplicity and its optimal performance in linear movement problems, the Kalman filter can substantially improve the precision of assigning subsequent positions to existing tracks [37,45]. Events such as cell division or migration out of and into the field of view, however, require higher level decisions such as initialization of new and termination of ending tracks. These behaviors necessitate processing on a symbolic level, as implemented by the Monitoring Module. As demonstrated, our system correctly initialized and accurately followed 85.48% of all valid tracks across all 5 image sequences. More importantly, the automatically determined average mean displacement values for the five cell populations did not show any significant differences from the estimated "true" rates, clearly outperforming the participants of the cell subset selection experiments.

Further improvements in the precision of the tracking process can potentially be achieved by consistently adapting the motion model in the Kalman Filter to the observed previous motion of the individual cell in each iteration [37]. Another option is to make use of Particle Filters (PF) [46], which have been applied to the area of multiple target tracking applications [28,47]. PF are able to deal with non-linearity of movement- and measurement-models, which enables more elaborate object state ahead prediction. Smal et al [28] have described the use of PF to track intracellular objects in fluorescence microscopic applications. The performance of their system was strongly dependent on signal-to-noise ratio (SNR) and object density. For a density of 40 objects per field of view, a SNR of at least 5 was required to reach an accuracy of 90%. As a general drawback, the computational cost of the PF framework increases considerably with the number of objects and particles used for motion prediction. Moreover, Godinez et al. [48] demonstrated in a similar experimental setting that Kalman Filters perform equal to Particle Filters under most conditions.

Alternatively, the use of Interacting Multiple Models (IMMs), which combine more than one motion predictor (e.g. Kalman Filter) to optimize state estimates, can be advantageous for modeling individual cell characteristics and cyclic cell behaviour [37]. Using an IMM with 4 interacting models for tracking of cells recorded with the phase contrast technique Li et al. report an accuracy of 77.8% - 88.9%. Genovesio et al [49] applied IMMs to the three dimensional tracking of fluorescent intracellular objects. In their evaluation, the IMM approach performed better than a single KF for different particle densities, but the differences in performance were small (ranging from 86.7% *vs*. 90% to 64.2% *vs*. 66.6% for lowest and highest particle densities, respectively). Moreover, the use of IMMs will lead to additional computational costs and requires a good *a-priori *knowledge of the cells behavior in order to select appropriate models, and/or the production of elaborate sets of training data for each individual cell population for estimating the increased number of model parameters (i.e. transition matrix probabilities).

In the current analysis, our system showed an effective processing time of 720 frames/h (framesize: 1024 × 1344 pixels) on an Intel Core 2 Duo, 2.4 GHz PC with 2 Gb RAM using a MatLab implementation.

## Availability and requirements

The software (TimeLapseAnalyzer) is available online http://www.informatik.uni-ulm.de/ni/staff/HKestler/tla/). A detailed documentation containing an in-depth description of the functionality of the software as well as example applications can be found in the supplementary information accompanying this article (Additional file 2) and on the project website.

**Project name: **TimeLapseAnalyzer

**Project home page: **http://www.informatik.uni-ulm.de/ni/staff/HKestler/tla/

**Operating system(s): **Platform independent

**Programming language: **MatLab (v. 7.2)

**Other requirements: **MATLAB Compiler Runtime (provided on the webpage if not available)

**License: **The source code is distributed under a Creative Commons Attribution-Noncommercial 3.0 License

**Any restrictions to use by non-academics: **n.a.

## Abbreviations

CI: Confidence Interval; DIC: Differential Interference Contrast; KF: Kalman Filter; MD/AMD: Mean Displacement/Average Mean Displacement; MM: Monitoring Module; spc: Sphingosylphosphorylcholine; UNN: Unique nearest neighbor; PF: Particle Filter; IMM: Interacting Multiple Models

## Authors' contributions

JH participated in the design of the study, implemented the software and helped to draft the manuscript. MB participated in the design, evaluated the procedures and helped to draft the manuscript. JMK and MS helped to revise initial versions of the manuscript and the tracking procedures. GvW provided image sequences and helped to draft the manuscript. DK provided image sequences and helped to draft the manuscript. TS provided overall direction and helped to draft the manuscript. TMG participated in its design and coordination and helped to draft the manuscript. HAK designed the study and drafted the manuscript. All authors read and approved the final manuscript.

## Supplementary Material

Additional file 1 Supplementary information: Core elements of the tracking system. Click here for file

Additional file 2 Supplementary information: Manual of the TimeLapseAnalyzer. Click here for file

Additional file 3 **Supplementary Video File A**. Example video file of automatically tracked untreated Panc1 cancer cells, recorded with the Differential Interference Contrast (DIC) imaging technique. Cell tracks (cell paths) are marked with colored spots. Green flashing spots indicate a cell division; red flashing spots indicate either a track loss or the leaving of a cell out of the field of view (event near the border). In addition, each track is also plotted into the last video frame for a final overview. Click here for file

Additional file 4 **Supplementary Video File B**. A second example video file of automatically tracked untreated Panc1 cancer cells, recorded with the DIC imaging technique. Cell tracks (cell paths) are marked with colored spots. Green flashing spots indicate a cell division; red flashing spots indicate either a track loss or the leaving of a cell out of the field of view (event near the border). In addition, each track is also plotted into the last video frame for a final overview. Click here for file

Additional file 5 **Supplementary Video File C**. Example video file of automatically tracked untreated Hela cancer cells, recorded with the Phase Contrast (PC) imaging technique. Cell tracks (cell paths) are marked with colored spots. Green flashing spots indicate a cell division; red flashing spots indicate either a track loss or the leaving of a cell out of the field of view (event near the border). In addition, each track is also plotted into the last video frame for a final overview. Click here for file

## References

[B1] Ayala R Shu T Tsai LH Trekking across the brain: the journey of neuronal migration Cell 2007 128 1 29 43 10.1016/j.cell.2006.12.021 17218253

[B2] Heng JI Nguyen L Castro DS Zimmer C Wildner H Armant O Skowronska-Krawczyk D Bedogni F Matter JM Hevner R Neurogenin 2 controls cortical neuron migration through regulation of Rnd2 Nature 2008 455 7209 114 118 10.1038/nature07198 18690213

[B3] Martin P Parkhurst SM Parallels between tissue repair and embryo morphogenesis Development 2004 131 13 3021 3034 10.1242/dev.01253 15197160

[B4] Schneider IC Haugh JM Mechanisms of gradient sensing and chemotaxis: conserved pathways, diverse regulation Cell Cycle 2006 5 11 1130 1134 16760661 10.4161/cc.5.11.2770

[B5] Henrickson SE Mempel TR Mazo IB Liu B Artyomov MN Zheng H Peixoto A Flynn MP Senman B Junt T T cell sensing of antigen dose governs interactive behavior with dendritic cells and sets a threshold for T cell activation Nat Immunol 2008 9 3 282 291 10.1038/ni1559 18204450 PMC2698867

[B6] Jacobelli J Bennett FC Pandurangi P Tooley AJ Krummel MF Myosin-IIA and ICAM-1 regulate the interchange between two distinct modes of T cell migration J Immunol 2009 182 4 2041 2050 10.4049/jimmunol.0803267 19201857

[B7] Tooley AJ Gilden J Jacobelli J Beemiller P Trimble WS Kinoshita M Krummel MF Amoeboid T lymphocytes require the septin cytoskeleton for cortical integrity and persistent motility Nat Cell Biol 2009 11 1 17 26 10.1038/ncb1808 19043408 PMC3777658

[B8] Rose DM Alon R Ginsberg MH Integrin modulation and signaling in leukocyte adhesion and migration Immunol Rev 2007 218 126 134 10.1111/j.1600-065X.2007.00536.x 17624949

[B9] Hanahan D Weinberg RA The hallmarks of cancer Cell 2000 100 1 57 70 10.1016/S0092-8674(00)81683-9 10647931

[B10] Schafer M Werner S Cancer as an overhealing wound: an old hypothesis revisited Nat Rev Mol Cell Biol 2008 9 8 628 638 10.1038/nrm2455 18628784

[B11] Michl P Ramjaun AR Pardo OE Warne PH Wagner M Poulsom R D'Arrigo C Ryder K Menke A Gress T CUTL1 is a target of TGF(beta) signaling that enhances cancer cell motility and invasiveness Cancer Cell 2005 7 6 521 532 10.1016/j.ccr.2005.05.018 15950902

[B12] Wolf K Wu YI Liu Y Geiger J Tam E Overall C Stack MS Friedl P Multi-step pericellular proteolysis controls the transition from individual to collective cancer cell invasion Nat Cell Biol 2007 9 8 893 904 10.1038/ncb1616 17618273

[B13] Witze ES Litman ES Argast GM Moon RT Ahn NG Wnt5a control of cell polarity and directional movement by polarized redistribution of adhesion receptors Science 2008 320 5874 365 369 10.1126/science.1151250 18420933 PMC3229220

[B14] Wels J Kaplan RN Rafii S Lyden D Migratory neighbors and distant invaders: tumor-associated niche cells Genes Dev 2008 22 5 559 574 10.1101/gad.1636908 18316475 PMC2731657

[B15] Dormann D Weijer CJ Visualizing signaling and cell movement during the multicellular stages of dictyostelium development Methods Mol Biol 2006 346 297 309 16957298 10.1385/1-59745-144-4:297

[B16] Dormann D Weijer CJ Imaging of cell migration EMBO J 2006 25 15 3480 3493 10.1038/sj.emboj.7601227 16900100 PMC1538568

[B17] Entschladen F Drell TL Lang K Masur K Palm D Bastian P Niggemann B Zaenker KS Analysis methods of human cell migration Exp Cell Res 2005 307 2 418 426 10.1016/j.yexcr.2005.03.029 15950622

[B18] Boldajipour B Mahabaleshwar H Kardash E Reichman-Fried M Blaser H Minina S Wilson D Xu Q Raz E Control of chemokine-guided cell migration by ligand sequestration Cell 2008 132 3 463 473 10.1016/j.cell.2007.12.034 18267076

[B19] Tang Q Adams JY Tooley AJ Bi M Fife BT Serra P Santamaria P Locksley RM Krummel MF Bluestone JA Visualizing regulatory T cell control of autoimmune responses in nonobese diabetic mice Nat Immunol 2006 7 1 83 92 10.1038/ni1289 16311599 PMC3057888

[B20] Blackman S Popoli R Design and Analysis of Modern Tracking Systems 1999 Norwood, MA, USA: Artech House Inc

[B21] Bar-Shalom Y Blair W Multitarget/Multisensor Tracking: Applications and Advances -- Volume III 2000 Norwood, MA, USA: Artech House Inc

[B22] Rasband WS ImageJ 1997 Bethesda, Maryland, USA: U. S. National Institutes of Health

[B23] Hajek J Sidak Z Theory of Rank Tests 1967 New York: Academic Press

[B24] Kalman RE A new approach to linear filtering and prediction problems Transactions of the ASME - Journal of Basic Engineering 1960 82 Series D 35 45

[B25] Grewal M Andrews A Kalman Filtering 1993 Englewood Cliffs, New Jersey, USA: Prentice Hall

[B26] Debeir O Milojevic D Leloup T Van Ham P Kiss R Decaestecker C Mitotic Tree Construction by Computer In VitroCell Tracking: a Tool for Proliferation and Motility Features Extraction Computer as a Tool, 2005 EUROCON 2005 The International Conference on. IEEE 2005 951 954 full_text

[B27] Bahnson A Athanassiou C Koebler D Qian L Shun T Shields D Yu H Wang H Goff J Cheng T Automated measurement of cell motility and proliferation BMC Cell Biology 2005 6 19 15831094 10.1186/1471-2121-6-19 PMC1097721

[B28] Smal I Draegestein K Galjart N Niessen W Meijering E Particle filtering for multiple object tracking in dynamic fluorescence microscopy images: application to microtubule growth analysis IEEE Trans Med Imaging 2008 27 6 789 804 10.1109/TMI.2008.916964 18541486

[B29] Machin M Santomaso A Mazzucato M Cozzi MR Battiston M Marco LD Canu P Single particle tracking across sequences of microscopical images: application to platelet adhesion under flow Ann Biomed Eng 2006 34 5 833 846 10.1007/s10439-006-9086-8 16708268

[B30] Centonze Frohlich V Phase contrast and differential interference contrast (DIC) microscopy J Vis Exp 2008 17 10.3791/844 PMC2762238 19066508

[B31] Gray AJ Young D Martin NJ Glasbey CA Cell identification and sizing using digital image analysis for estimation of cell biomass in High Rate Algal Ponds J Appl Phycol 2002 14 193 204 10.1023/A:1019976310527

[B32] Kam Z Microscopic differential interference contrast image processing by line integration (LID) and deconvolution Bioimaging 1998 6 166 176 10.1002/1361-6374(199812)6:4<166::AID-BIO2>3.0.CO;2-Y

[B33] Meijering E Smal I Dzyubachyk O Olivo-Marin JC Time-Lapse Imaging Microscope Image Processing 2008 Academic Press 401 440 full_text

[B34] Simon I Pound CR Partin AW Clemens JQ Christens-Barry WA Automated Image Analysis System for Detecting Boundaries of Live Prostate Cancer Cells Cytometry 1998 31 4 287 294 10.1002/(SICI)1097-0320(19980401)31:4<287::AID-CYTO8>3.0.CO;2-G 9551604

[B35] Young D Glasbey CA Gray AJ Martin NJ Towards automatic cell identifcation in DIC microscopy Journal of Microscopy 1998 192 186 193 10.1046/j.1365-2818.1998.00397.x 9853375

[B36] Wu K Gauthier D Levine DM Live cell image segmentation IEEE Trans Biomed Eng 1995 42 1 1 12 10.1109/10.362924 7851922

[B37] Li K Miller ED Chen M Kanade T Weiss LE Campbell PG Cell population tracking and lineage construction with spatiotemporal context Med Image Anal 2008 12 5 546 566 10.1016/j.media.2008.06.001 18656418 PMC2670445

[B38] Sacan A Ferhatosmanoglu H Coskun H CellTrack: an open-source software for cell tracking and motility analysis Bioinformatics 2008 24 14 1647 1649 10.1093/bioinformatics/btn247 18511469

[B39] Chan F Vese LA Active Contour Without Edges IEEE Trans Image Proc 2001 10 2 266 277 10.1109/83.902291 18249617

[B40] Yilmaz A Shah M Contour Based Object Tracking with Occlusion Handling in Video Acquired Using Mobile Cameras IEEE Trans PAMI 2004 26 11 1531 1536 10.1109/TPAMI.2004.96 15521500

[B41] Hamahashi S Onami S Kitano H Detection of nuclei in 4D Nomarski DIC microscope images of early Caenorhabditis elegans embryos using local image entropy and object tracking BMC Bioinformatics 2005 6 125 15910690 10.1186/1471-2105-6-125 PMC1175842

[B42] Beucher S Lantuejoul C Use of Watersheds in Contour Detection Int Workshop on Image Processing, Real-Time edge and motion detection/estimation 1979 132 Rennes, France: IRISA 2.1 2.12

[B43] Chen X Zhou X Wong ST Automated segmentation, classification, and tracking of cancer cell nuclei in time-lapse microscopy IEEE Trans Biomed Eng 2006 53 4 762 766 10.1109/TBME.2006.870201 16602586

[B44] Yan J Zhou X Yang Q Liu N Cheng Q Wong STC An Effective System for Optical Microscopy Cell Image Segmentation, Tracking and Cell Phase Identification International Conference on Image Processing (ICIP) 2006 Atlanta: IEEE 1917 1920

[B45] Althoff K Degerman J Gustavsson T Kalviainen H, Parkkinen J, Kaarna A Combined Segmentation and Tracking of Neural Stem-Cells Image Analysis, 14th Scandinavian Conference on Image Analysis, SCIA 2005 2005 3540 Berlin: Springer 282 291

[B46] Ristic B Arulampalam S Gordon N Beyond the Kalman Filter: Particle Filters for Tracking Applications 2004 Artech House

[B47] Shen H Nelson G Kennedy S Nelson D Johnson J Spiller D White MRH Kella DB Automatic tracking of biological cells and compartments using particle filters and active contours Chemometrics and Intelligent Laboratory Systems 2006 82 1-2 276 282 10.1016/j.chemolab.2005.07.007

[B48] Godinez WJ Lampe M Worz S Muller B Eils R Rohr K Deterministic and probabilistic approaches for tracking virus particles in time-lapse fluorescence microscopy image sequences Med Image Anal 2009 13 2 325 342 10.1016/j.media.2008.12.004 19223219

[B49] Genovesio A Liedl T Emiliani V Parak WJ Coppey-Moisan M Olivo-Marin JC Multiple particle tracking in 3-D+t microscopy: method and application to the tracking of endocytosed quantum dots IEEE Trans Image Process 2006 15 5 1062 1070 10.1109/TIP.2006.872323 16671288

