# Supplementary material for: Significantly improved precision of cell migration analysis in time-lapse video microscopy through use of a fully automated tracking system
Source: BMC Cell Biol. 2010 Apr 8;11:24. doi: 10.1186/1471-2121-11-24 (PMC2858025; doi:10.1186/1471-2121-11-24)
Supplement: Additional file 1 — Supplementary information: Core elements of the tracking system. [file 1471-2121-11-24-S1.PDF]

Significantly improved precision of cell migration analysis in time-lapse video microscopy through use of a fully automated tracking system

Supplementary information: Core elements of the tracking system

Johannes Huth[1,3,*], Malte Buchholz[3,*], Martin Schmucker[1], Götz von Wichert[2], Denis Krndija[2], Thomas Seufferlein[2,4], Thomas M Gress[3,+] and Hans A Kestler[1,2,+]

1: Neural Information Processing, University of Ulm, Germany
2: Internal Medicine I - Gastroenterology, University Hospital Ulm, Germany
3: Department of Gastroenterology and Endocrinology, University Hospital of Marburg, Germany
4: Department of Internal Medicine I, Martin-Luther-University, Halle-Wittenberg, Germany
*: These authors share first authorship
+: These authors share last authorship

1 Introduction

This document summarizes the core elements of the automated tracking system used in the paper "A fully automated tracking system to significantly improve the precision of cell migration analysis in time-lapse video microscopy". The system implements data association tracking and is comprised of three parts, namely cell centroid extraction, motion filtering, and track monitoring.

The systems data input consists of the geometrical centroids of cells which have to be extracted from microscopic recordings in each iteration (i.e. each new recorded frame). The required image processing routines are outlined in section 3.
A *Kalman Filter*(KF) was used as motion filter to follow single objects (cells) through an image sequence and optimize the objects state estimates. The implementation is introduced in more detail in section 4. As the KF requires measurements for the update step in each iteration, the *unique nearest neighbor* (UNN) algorithm is introduced (see section 5).
Higher level events like cell division or moving of cells out and into the field of view frequently occur in live cell tracking experiments and have to be supervised accordingly, as they are beyond the scope of the KF. The solution is to use a supervising unit which we refer to as *monitoring module* (MM). This unit is explained in section 6.

2 Cell migration rates

The migration rate of cells is commonly measured via the mean displacement (MD, i.e. the mean distance (μm) traveled per minute) of the cell centroid: Let the recording time from frame to frame be t, N the number of frames and d_i the euclidean distance a cell travels between frame i and frame $i+1$ (in μm). The MD of the cell c_i is the expectation over all displacements:

$$\mathrm{MD}(c_i) = \frac{1}{N-1}\sum_{i=1}^{N-1} d_i t^{-1}$$

The migratory potential of cell populations is expressed as the average mean displacement (AMD) of all cells

in the analysis. The AMD of a cell population S with $|S| = M$ cells is computed as

$$\text{AMD(S)} = \frac{1}{M}\sum_{i=1}^{M}\text{MD}(c_i)$$

3 Cell centroid extraction

To extract the geometrical cell centroids from each frame, two binary masks were computed, see figure 1. The first "coarse" mask B_C denotes general cell regions, without separating individual cells from each other. The second mask B_D represents details in individual cells. Both masks were subsequently combined to define individual cell centroids whilst omitting noise in the media.
The extraction of B_C was mainly based on the structural information of the cell tissue. At first to speed up the process, each frame was scaled by factor 0.4. Then the local image entropy in a (7×7) neighborhood was computed to highlight cell regions. This entropy image was multiplied by a median filtered (25×25) [Jähne, 2005] version of the original image to delete small noise regions, such as parts of dead cells in the media. Image binarisation was performed using Otsu thresholding [Otsu, 1979] and subsequent dilation (5×5 mask) [Jähne, 2005]. As a last step, regions which are too small (< 1000 pixels) to represent living cells were deleted.
For B_D, the illumination intensity was the feature of choice: each frame was smoothed by a median filter (21×21). For the binarisation a sensitive local thresholding technique, extended maxima transform [Soille, 2003], was applied on the smoothed image and afterwards the geometrical centroids of all regions computed.

B_C and B_D were combined by pixel-wise multiplication to the binary image B to delete noise particles in non-cell regions. Only those centroids from B_D lying within a cell region of B_C were therefore further processed in the consecutive steps by shifting them toward their regional local maxima using a gradient based centroid shifting algorithm. Centroids that were shifted into non-cell tissue regions (defined by $-B_C$) were also deleted. Shifted centroids were merged if they were closer to each other than 25 pixels.

To propagate centroids steadily through the image sequence, an additional backup mechanism was used. The centroid image C from one previous processed frame was combined with the centroid image B from the actual frame $(B + C)$ before the centroid shifting was applied. The centroid extraction for one frame takes approximately five seconds on our system (image size: 1344×1024).

4 Kalman Filter implementation

Kalman Filter, first introduced by R.E. Kalman [Kalman, 1960], can be used in object tracking to optimize the estimate $\hat{\mathbf{s}}_t$ of an unknown real object state $\mathbf{s}_t$ using erroneous measurements and a state ahead prediction in each iteration. Given an optimized past state estimate $\hat{\mathbf{s}}_{t-1}$ at time $t-1$ the filter predicts the possible future of the object $\mathbf{s}_t$ as

$$\mathbf{s}_t = \mathbf{A}\hat{\mathbf{s}}_{t-1} + \mathbf{w} \tag{1}$$

where $\mathbf{A}$ is an underlying movement model. The predicted state is compared to an actual noisy measurement $\mathbf{z}_t$ and the estimation of the real objects state is optimized using a weighted difference between $\mathbf{z}_t$ and $\mathbf{s}_t$

$$\hat{\mathbf{s}}_t = \mathbf{s}_t + \mathbf{K}(\mathbf{z}_t - \mathbf{s}_t) \tag{2}$$

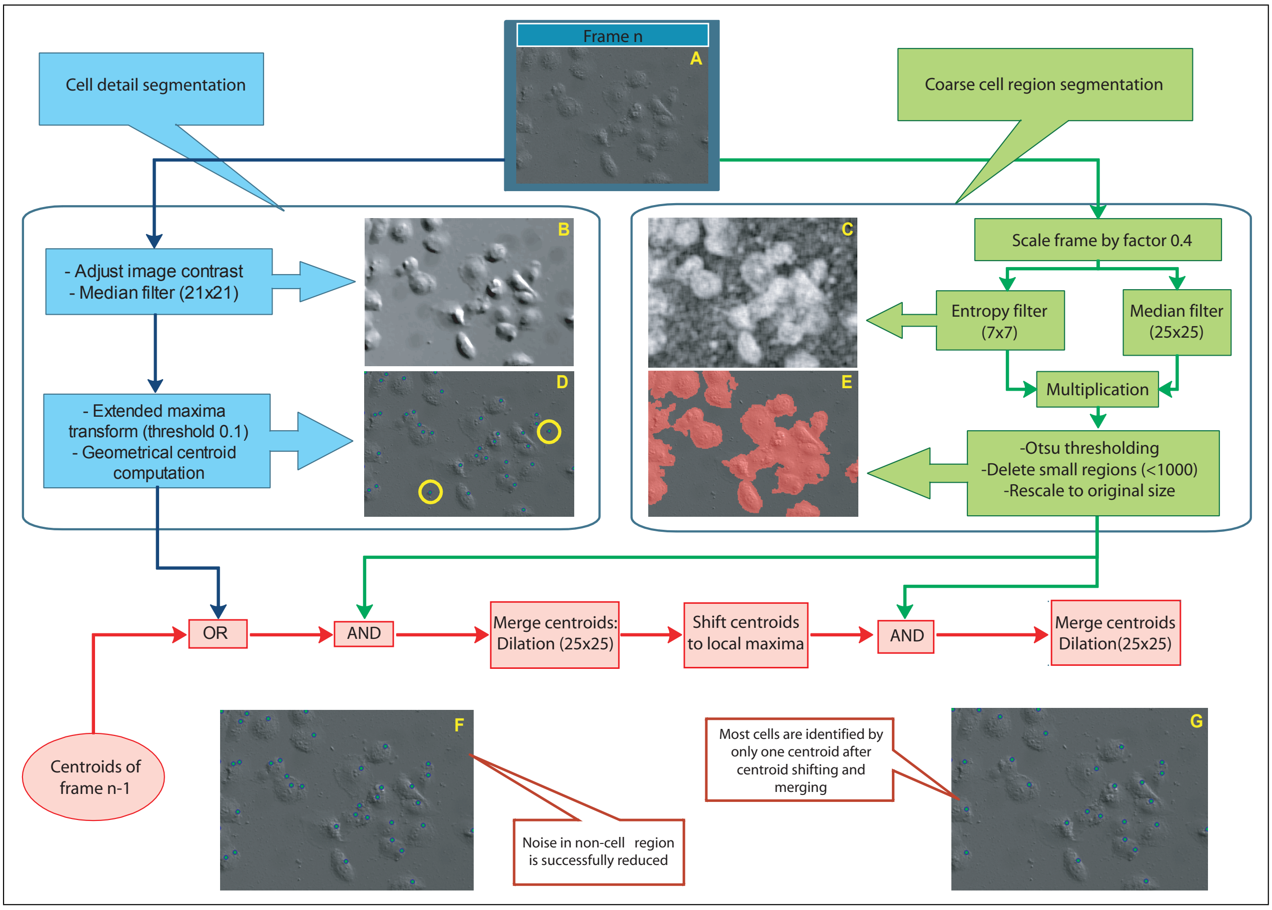

Figure 1: Schematic work-flow and examples of intermediary steps of cell centroid extraction from microscopic images. Each new frame (A) will be processed in two distinct steps, namely cell detail segmentation (left, blue box) and cell region segmentation (right, green box). The detected centroids from the detail segmentation are first combined with the extracted centroids of one past frame to propagate cell centroids steadily through an image sequence. Afterwards the combination of the cell region image and the cell centroid image leads to deletion of cell positions in non-cell regions (panel F). Subsequent centroid merging and shifting finally concentrate groups of possible centroids within one cell to form a single cell centroid (panel H).

The noisy (erroneous) measurement $\mathbf{z}_t$ is defined as

$$\mathbf{z}_t = \mathbf{H}\mathbf{s}_t + \mathbf{v} \tag{3}$$

$\mathbf{K}$ is known as the *Kalman Gain (KG)*, $\mathbf{H}$ relates the state to the measurement. $\mathbf{w}$ and $\mathbf{v}$ are additional Gaussian distributed system error and measurement error, respectively.

The discrete KF algorithm now consists of two alternating steps: prediction and correction.
In the prediction step at time $t-1$, the filter makes an assumption about the future state of the system at time t following equation 1. In the correction step, an optimized (*a posteriori*) state estimate is computed using the measurement $\mathbf{z}_{t+1}$, the *a priori* state estimate $\mathbf{s}_t$ and the weight matrix $\mathbf{K}$ (equation 2). The residual between the *a priori* and *a posteriori* state is subsequently used to update the Kalman Gain $\mathbf{K}$.
The KG weights the residual of the measurement $\mathbf{z}_t$ and the predicted state $\mathbf{s}_t$. The weighing is in general dependent on two factors, namely the quality of the movement model and the measurement error covariance. If the movement model agrees well with the real cell movement, the weights will be small, so that the entire system relies more on the movement model than on the noisy measurement. If on the other hand the system

error is high, the model is trusted less and the state optimization relies more on the actual measurement (see equation 2).
Also if the measurement error is high, the weights will be small and vice versa. As the measurement error covariance was fixed in our system, the update of $\mathbf{K}$ was only related to the agreement between the movement model and the real cell behaviour.

We realized the state vector of an object (cell) as $\mathbf{s}_i^j = (x_i^j, x_{i-1}^j, y_i^j, y_{i-1}^j,)^T$, belonging to the j-th track in frame i. The movement model and the measurement relation matrix were defined as

$$\mathbf{A} = \begin{pmatrix} 2 & -1 & 0 & 0 \\ 1 & 0 & 0 & 0 \\ 0 & 0 & 2 & -1 \\ 0 & 0 & 1 & 0 \end{pmatrix}, \qquad \mathbf{H} = \begin{pmatrix} 1 & 0 & 0 & 0 \\ 0 & 0 & 1 & 0 \end{pmatrix}$$

modeling the fact, that consecutive cell displacements are likely to be comparable in magnitude and direction. To identify the process noise variance introduced by this basic assumption, we computed the x- and y-displacement series of 420 manual extracted and smoothed track $\mathbf{G}^j$ (tracks were smoothed using a centered moving average filter with two previous and two past values). The displacement series for the individual x and y component are computed as

$$\begin{aligned} \mathbf{d_x}^j &= \{(x_2^j - x_1^j), (x_3^j - x_2^j), \cdots, (x_N^j - x_{N-1}^j)\} \\ &= \{\mathbf{d_x}^j(1), \mathbf{d_x}^j(2), \cdots, \mathbf{d_x}^j(N-1)\} \\ \mathbf{d_y}^j &= \{(y_2^j - y_1^j), (y_3^j - y_2^j), \cdots, (y_N^j - y_{N-1}^j)\} \\ &= \{\mathbf{d_y}^j(1), \mathbf{d_y}^j(2), \cdots, \mathbf{d_y}^j(N-1)\} \end{aligned}$$

If the model A were in perfect agreement with the real cell movement, consecutive displacement values (e.g. $\mathbf{d_x}^j(i+1), \mathbf{d_x}^j(i)$) would be equal to each other. While perfect agreement is extremely improbable, we expect the differences of consecutive displacement values to be located around a zero mean if the model is appropriate. The standard deviations can than be used to model the process (movement model) uncertainty. We therefore computed the set D of differences between consecutive displacements as

$$D = D_x \cup D_y \tag{4}$$

$$D_x = \{\mathbf{d_x}^j(i+1) - \mathbf{d_x}^j(i) \mid i = 1 \cdots N-1, j = 1 \cdots \|G\|\} \tag{5}$$

$$D_y = \{\mathbf{d_y}^j(i+1) - \mathbf{d_y}^j(i) \mid i = 1 \cdots N-1, j = 1 \cdots \|G\|\}$$

and found that the the histogram reveals a zero mean and a standard deviation of $\sigma \approx 2$ pixel. Given this information and the fact that the analysis was based on the manually extracted, and thus error prone, tracking data, the system error was modeled with a slightly higher standard error by ($w \sim \mathrm{N}(0, \sqrt{5})$). The measurement noise v was modeled as ($v \sim \mathrm{N}(0, 5)$) with a standard deviation of five pixels, taking the possibility of inaccurate segmentation results into account. Figure 2 demonstrates the "denoising" effect of Kalman filtering with the previously defined settings on a cell track artificially superimposed with noise (7.5 μm deviation).

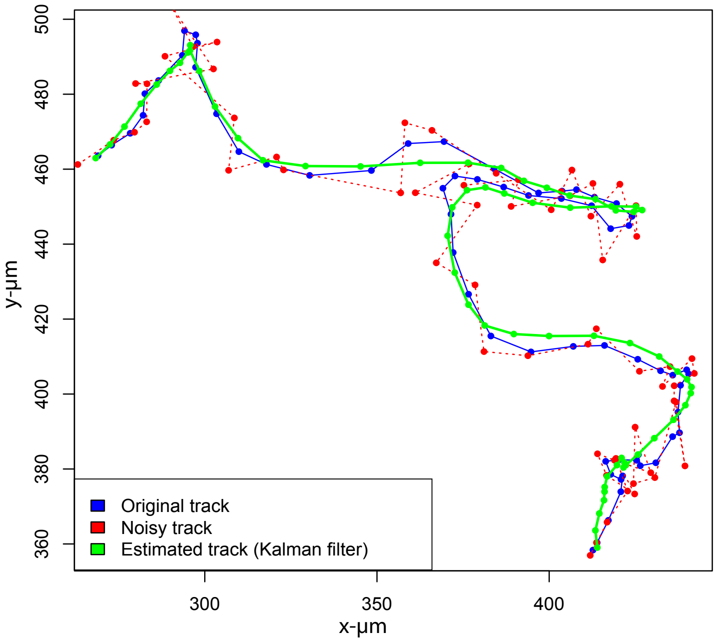

Figure 2: Kalman filter tracked cell path. The blue line displays the original cell path without any influence of noise. The red dots indicate the noisy measurements, which were varied within a standard deviation of five pixel around the original (blue) path. The dashed red line shows the track that would result from taking the noisy measurements as real centroid positions. The track varies strongly around the original blue track. The green line displays the track derived by the Kalman Filter implemented in this project. A main part of the noise is successfully filtered with our approach so that the Kalman track appears much smoother than the track from the noisy measurement. Note that the KF with constant velocity model also performs well at major turning points of the trajectory.

5 Unique nearest neighbor algorithm

Let $\mathcal{A} = \{a_1 \cdots a_N\}$ be the set of centroids of the N track endings, let $\mathcal{B} = \{b_1 \cdots b_M\}$ be the set of segmented M cell centroids in the actual regarded frame (i.e. b is equivalent to z in equation 3). The introduced iterative algorithm tries to associate an unique nearest neighbor from $\mathcal{B}$ to each of the N centroids from $\mathcal{A}$. Uniqueness is defined by the fact that a new centroid b_j is assigned to one and only one track end a_i.

To initialize the algorithm, a set of nearest neighbors is computed for all entries in $\mathcal{A}$ using a *kd-search tree* [de Berg et al., 1997]. This pre-selection is called *gating* and is common procedure in tracking applications to identify "reasonable" observation-to-track pairings [Blackman and Popoli, 1999]. The search tree is build from the set $\mathcal{B}$ and requires $O(M \; log \; M)$ building time. A rectangular (squared) search query can be done in $O(\sqrt{M} + k)$ time. The search range is given by the actual regarded track end a_i (center of the search square) and a predefined maximum distance $d_{\max}$ which in our case is equal for x and y direction.
The result for each query is a sorted set $\mathcal{B}^{a_i}$ of q neighbors belonging to the i-th centroid a_i:

$$\begin{aligned} \mathcal{B}^{a_i} &= \{b_1^i \cdots b_q^i\} \\ \text{with} \quad & \mathcal{B}^{a_i} \subseteq \mathcal{B} \\ \text{and} \quad & d(a_i, b_j^i) \leq d(a_i, b_{j+1}^i), \forall b_j^i \in \mathcal{B}^{a_i} \end{aligned}$$

where $d(a, b)$ is the euclidean distance between a and b. These sets are further restricted by defining a maximum number k of nearest neighbors *and* by omitting all centroids whose euclidean distance from the

query point a_i is beyond $d_{\max}$:

$$\begin{aligned} d(a_i, b_r^i) &\leq d(a_i, b_j^i),\ \forall b_j^i \in \{\mathcal{B} \cap \mathcal{B}^{a_i}\}, \\ \text{with} & \quad r = ||\mathcal{B}^{a_i}||,\ r \leq k \\ \text{and} & \quad d(a_i, b_r^i) \leq d_{\max} \end{aligned}$$

Thus the set $\mathcal{B}^{a_i}$ contains at most k centroids from $\mathcal{B}$ which are sorted regarding to their distance to a_i. This distance is less or equal to $d_{\max}$. Note that it is possible that the sets $\mathcal{B}^{a_i}$ are of different size or even empty.

After all sets $\mathcal{B}^{a_i}$ are computed, an array R of size N and the result list L is initialized. R will store lists of indices of requested but already reserved nearest neighbors, L stores accepted associations. The list of remaining unassociated measurement is later referred to as $\mathcal{B}^R$. The next operations outline our UNN algorithm.

1. Set $\mathcal{B}^R \leftarrow \mathcal{B}$
2. Find the index of the centroid from $\mathcal{A}$ with the closest nearest neighbor as:
   $i = \underset{i}{\operatorname{argmin}}(d(a_i, \mathcal{B}_1^{a_i}))$. Let the corresponding best matching pair be $\hat{a}_i$ and $\hat{b}_j$.
3. Make the assignment $\hat{a}_i \rightarrow \hat{b}_j$ and store it in the result list L; delete $\hat{a}_i$ from $\mathcal{A}$,delete $\hat{b}_j$ from $\mathcal{B}^R$ and delete/clear $\mathcal{B}^{\hat{a}_i}$.
4. $\forall$ remaining (non empty) $\mathcal{B}^{a_i}, i \neq \hat{i}$: **If** $\hat{b}_j \in \mathcal{B}^{a_i}$ **delete** $\hat{b}_j$ from the set ($\mathcal{B}^{a_i} \leftarrow \mathcal{B}^{a_i} \backslash \hat{b}_j$).
   Get the list $l \leftarrow R[i]$ \\ *l may be empty*
   Append $\hat{b}_j$ to l together with a pointer on a_i
   Store l in $R[i]$
5. **If** either all $\mathcal{B}^{a_i}$ or $\mathcal{A}$ is empty, sort each list l_i in R and return R, $\mathcal{B}^R$ and L
   else return to **2**.

It is essential that in each iteration only the best NN connection is chosen and accepted. The closest and thus most likely track-measurement pairs are privileged and accepted first. The algorithm runs steps 2-5 $O(N)$ times. The time for finding the minimum element (step 2) is $O(N)$. The time for searching for equal elements in step 4 is $O(k \cdot N)$. The overall running time is therefore determined as $O(N^2)$. Figure 3 illustrates a case where our UNN-algorithm performs superior to the naive NN approach.

6 Monitoring Module

After assigning a track end a to a measurement b, ($a \rightarrow b$), the euclidean distance between the two points ($\epsilon = d(a, b)$) is used as the reference value in the MM. Based on predefined thresholds, the MM decides about possible states of the cell. i.e. if it takes part in a mitosis event, or if the cell is in the process of leaving the field of view. The entire decision process is outlined in figure 4. It uses two main thresholds T_2 and T_1 where T_2 denotes the maximum physically possible distance a cell is able to travel in the given time interval (strongly dependent on cell type). In our experiments, T_1 was defined as $T_1 = T_2/2$.

Mitosis

If $\epsilon \leq T_1$, the assignment ($c \rightarrow b$) is accepted and b is used to update the state of a in the KF. If $\epsilon > T_1$, the possibility of a mitotic event is considered. For this purpose, we use the lists $l_i = R[i]$ from the UNN

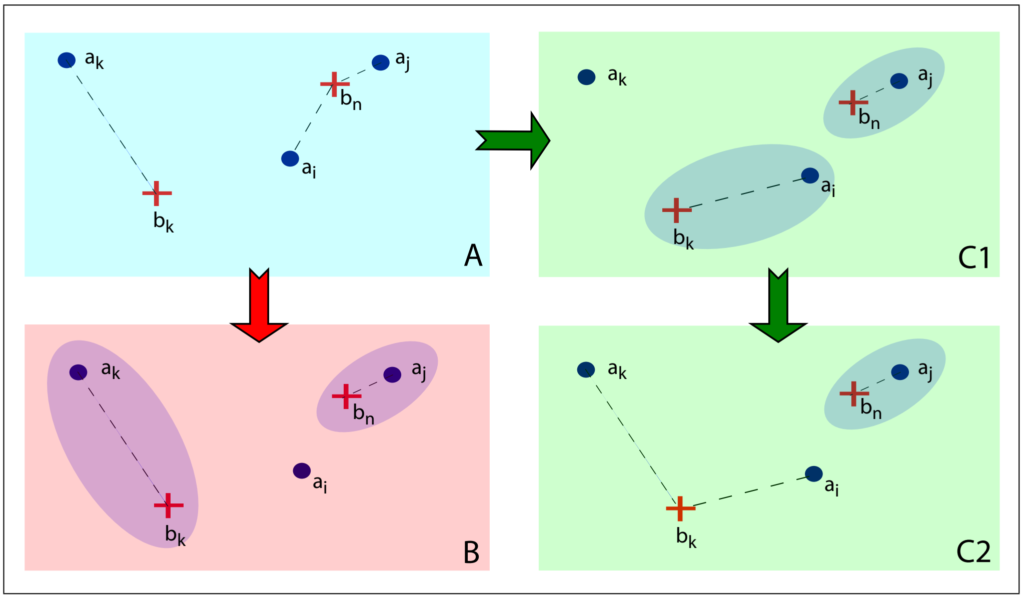

Figure 3: Illustration of the UNN algorithm. Dots mark track ends and crosses mark measurements. In this example, the NN of a centroid a_i is b_n. But b_n is also the NN to a_j, and a_j is closer to b_n than a_i. Also in this iteration, a single association is found between a centroid a_k and b_k (see image A; note that in each image only the connections between each a_i and b_1^i, i.e. the first nearest neighbor is illustrated). Acceptance of the single association $a_k \rightarrow b_k$ in addition to the best matching pair (here (a_j, b_n)) would result in image B (the ellipses mark an accepted association, centroids in the ellipse are not further processed). Thus the correct association $a_i \rightarrow b_k$ would not be found. In contrast, if only the best matching pair is accepted, the next iteration starts with the situation outlined in image C1. The association between a_j and b_n is valid and accepted and not further regarded. When the remaining centroids are further processed, the next closest pair is (a_i, b_k). Therefore the association $a_i \rightarrow b_k$ is accepted, and a_k remains unassociated in this case, which is the desired result. The algorithm will then terminate, as all the lists $\mathcal{B}^{a_i}$ are empty.

algorithm. From each list l, information about whether a was situated closer to another measurement which was already reserved for the assignment to another track end a_2 is extracted (see e.g. figure 3 where $R[i]$ will contain the index of b_n and a link to a_2). If this is the case, the distance between a and a_2 is validated (in figure 3, this would be the distance between a_i and a_j). If it lies below a predefined mitosis threshold, the track pair (a, a_2) is marked for a possible mitosis event. If the distance lies beyond the threshold, the next centroid in l is tested. The threshold is dependent on the cell size and the cells' growth behaviour (single, or in colonies) and has to be adjusted accordingly. If a cell is marked as mitotic with the same partner cell over a predefined number of frames, the track terminates.

Cell Leaving event

In the case that there is no possible partner track or the partner track is not sufficiently close to a, the track belonging to a is checked for a border leaving event. This is again dependent on a threshold distance to the frame border. If the distance lies below the threshold, the track is marked as a possible border track.
If $T_1 < \epsilon \leq T_2$ and mitosis or leaving events did not lead to track termination, the measurement b is still used partly in the KF update. We don't "trust" b entirely to be the real next measurement, but we keep the general signal direction. Only the amplitude is adjusted as $\bar{b} = a + \frac{(b-a)}{\sqrt{\epsilon}}$ and $\bar{b}$ is used in the KF update. In the case that $\epsilon > T_2$ and no mitosis or cell leaving event was detected, the track is marked as possibly lost. In this case, the measurement b will not be taken for the KF update. Instead, the track end a is taken again for the update step.

Track initialization

If the set $\mathcal{B}^R$ of remaining unassociated measurements is not empty, track initialisation is performed. If a measurement $b_j \in \mathcal{B}^R$ lies closer to the frame border than the predefined border threshold, a new track is initialized.[1] New cell tracks are only initialized at the border of the frame, since we used backward tracking [Debeir et al., 2004] in our application, so that cell mitosis will be perceived as cell merging. In our current evaluation, all cell tracks were initialized in the starting frame (last frame). During the course of a tracking analysis, new track initialization was only accepted at the frame border.

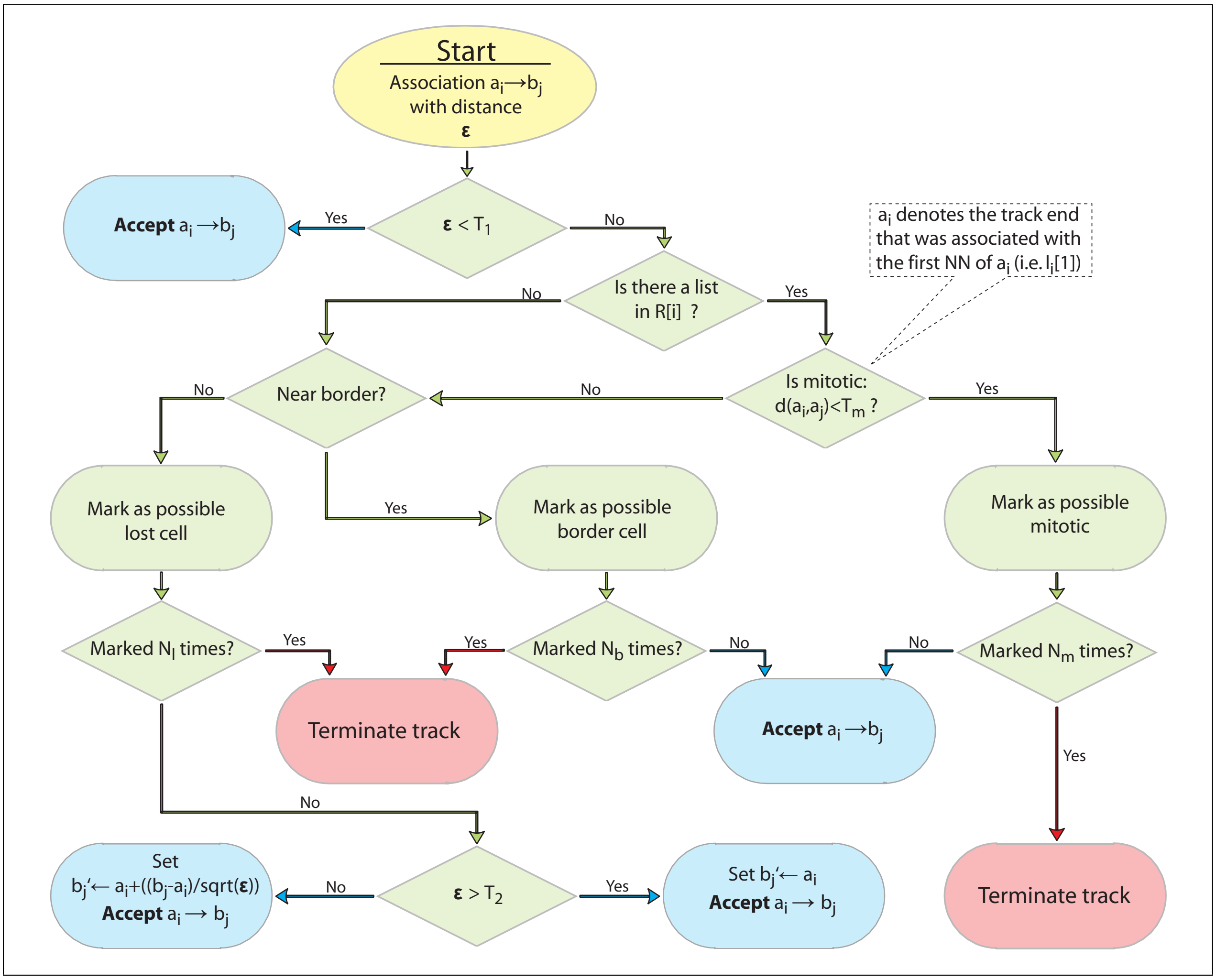

Figure 4: Work flow of the Monitoring Module to discover "higher level" cell states like *Mitotic*, *Leaving* or *Lost*. The monitoring starts with a low level data association ($a_i \rightarrow b_j$) from the UNN algorithm (yellow ellipse). The validation is done for each data association in the UNN result list L individually. If the actual track-measurement-pair is accepted (blue labeled boxes), it will be further processed in the KF to optimize the object state estimate. If the pair is not accepted directly, the track either terminates (red labeled boxes) or the measurement is adjusted before the next KF update (orange labeled boxes).

References

[Blackman and Popoli, 1999] Blackman, S. and Popoli, R. (1999). *Design and Analysis of Modern Tracking Systems*. Artech House.

[1] Please Note:
Due to its relative independence, track initialization is not included in the decision process illustration (figure 4). Track initialization is scheduled between track association and KF-updates.

[de Berg et al., 1997] de Berg, M., van Kreveld, M., and Overmars, M. (1997). *Computational Geometry, Algorithms and Applications*. Springer.

[Debeir et al., 2004] Debeir, O., Camby, I., Kiss, R., Van Ham, P., and Decaestecker, C. (2004). A model-based approach for automated in vitro cell tracking and chemotaxis analyses. *Cytometry Part A*, 60A(1):29–40.

[Jähne, 2005] Jähne, B. (2005). *Digitale Bildverarbeitung*. Springer-Verlag Berlin Heidelberg.

[Kalman, 1960] Kalman, R. E. (1960). A new approach to linear filtering and prediction problems. *Transactions of the ASME - Journal of Basic Engineering*, 82(Series D):35–45.

[Otsu, 1979] Otsu, N. (1979). A threshold selection method from gray-level histograms. *IEEE Transactions on Systems, Man and Cybernetics*, 9(1):62–66.

[Soille, 2003] Soille, P. (2003). *Morphological Image Analysis: Principles and Applications*. Springer-Verlag New York, Inc., Secaucus, NJ, USA.
