# Supplementary material for: Significantly improved precision of cell migration analysis in time-lapse video microscopy through use of a fully automated tracking system
Source: BMC Cell Biol. 2010 Apr 8;11:24. doi: 10.1186/1471-2121-11-24 (PMC2858025; doi:10.1186/1471-2121-11-24)
Supplement: Additional file 2 — Supplementary information: Manual of the TimeLapseAnalyzer. [file 1471-2121-11-24-S2.PDF]

TLA
Time Lapse Analyzer
A brief manual

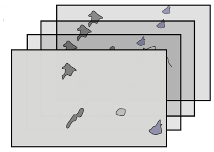

Johannes Huth
Johann M Kraus
Hans A Kestler

May 20, 2009

Contents

1. Introduction - about this document **4**

2. Graphical overview - GUI advanced view map **5**

3. Installing and running the TLA **7**
3.1. License . . . 7
3.2. Installation errors . . . 7

4. Basic user mode **7**
4.1. Running a time lapse analysis . . . 7
4.2. Program sample . . . 8
4.3. Useful functions . . . 10
4.4. The preview panel . . . 12

5. Menus **13**
5.1. File . . . 13
5.2. Setup . . . 14
5.3. Options . . . 15
5.4. Tools . . . 16
5.5. Help . . . 18

6. Advanced setup construction mode **18**
6.1. Quickoptions . . . 19
6.2. Test the image processing . . . 19

7. Workstack - defining image processing work flow **20**
7.1. Expressions, Units, Operations . . . 20
7.2. General process flow . . . 21
7.3. Usefor - a basic operator . . . 22
7.4. Complexity -he '#'-perator . . . 23
7.5. Convenient use of the workstack - compression of lines . . . 25
7.6. WS examples . . . 27
7.6.1. Mean stack image . . . 27
7.6.2. Cell tissue filter . . . 27
7.7. Workstack buttons and context menues . . . 27
7.7.1. Context menu . . . 29

8. Time lapse functions **29**
8.1. Frame selection . . . 30
8.2. Cell tracking - multi target tracking . . . 31
8.3. Image batch-processing . . . 33
8.4. Movies . . . 33
8.5. Alter and test time lapse settings . . . 33

9. Cell tracking result viewer **35**
9.1. Result list . . . 35
9.1.1. Working with entries . . . 35
9.2. Plotting results . . . 36
9.2.1. Plot types . . . 36
9.2.2. Marker settings . . . 37

9.2.3. Result subsets . . . 37
9.2.4. Zoom and image display . . . 37
9.2.5. Open separate figures . . . 37
9.3. Video options . . . 38
9.3.1. Files and directories . . . 38
9.3.2. Image processing options . . . 38
9.3.3. Track and video settings . . . 38
9.3.4. Batch processing files . . . 39
9.4. Menus . . . 40
9.4.1. File . . . 40
9.4.2. Plotting . . . 40
9.4.3. Help . . . 41

10. Interface for MatLab image processing functions 42

11. Further informations for MatLab developer 42

A. WS-function listing 43
A.1. Image processing . . . 43
A.2. Segmentation and binarisation . . . 47
A.3. Postprocessing . . . 47

B. Preparing setups for public use 48

1. Introduction - about this document

This is the documentation version for the Time Lapse Analyser (TLA), a free, cross-platform, open source software for high throughput time-lapse data processing for live cell imaging.

TLA is a graphical tool which enables easy access to high-throughput live cell imaging for every user, regardless of the individual expertise. Beginners can easily process stacks of time-lapse data by loading an appropriate image processing setup and time-lapse evaluation setup. Advanced users can modify existing setups, or create new setups which suit their need and special experimental conditions.

Using the TLA one can choose between the basic user mode and the advanced user mode. In the basic mode one can only load and use the setups, in the extended user mode, one can also modify and create setups. Using setups and thus running the TLA is simple and the work flow is explained in the section 4. An overview over setup construction and modification is provided in section 6.

Most functions and menu entries are simply listed and explained in a lookup-style and for convenience hyper-links are used. This makes this document a look-up table more than a read-from-top-to-bottom instruction. However, some section will explain general concepts which will provide an easy access to the entire functionality of the TLA.

The first section includes a graphical map of the TLA with enumerated panels, functions, buttons, and menu entries. Use the hyper-links to find the corresponding section in the document.

If there are any questions, please do not hesitate to write the authors. We are thankful for all comments on our program as we constantly trying to improve it. Contact information can be found on the projects homepage: TLA homepage.

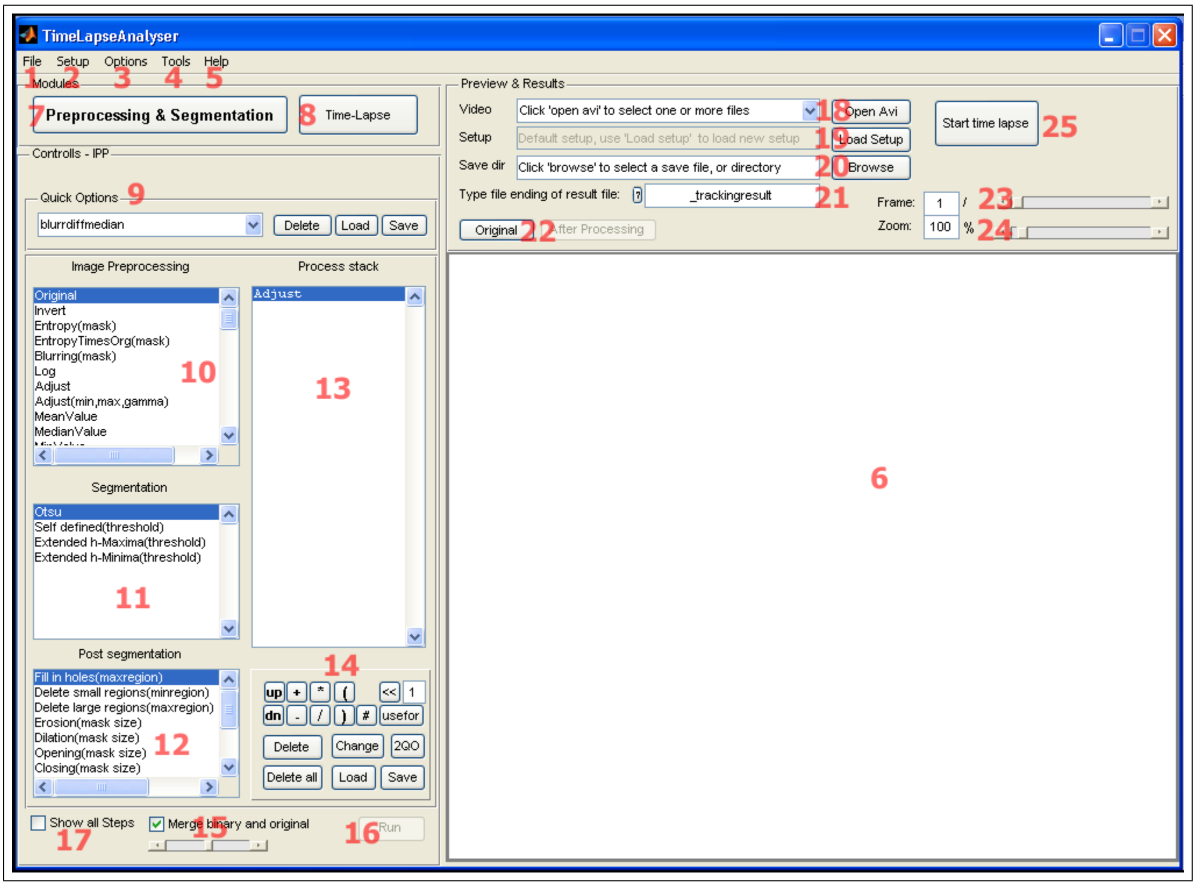

2. Graphical overview - GUI advanced view map

1: Menu File (5.1)
2: Menu Setup (5.2)
3: Menu Options (5.3)
4: Menu Tools (5.4)
5: Menu Help (5.5)
6: Preview panel (4.4)
7: Image processing panel (6)
8: Time lapse panel (8)
9: Quick options (6.1)
10: Image processing options (6)
11: Segmentation/ binarisation (6)
12: Post processing options (6)
13: Workstack (7)
14: Workstack instruments (7.7)
15: Merge images (6)
16: Run single image processing (6.2)
17: Show all images checkbox (6)
18: Open avi files (4.1)
19: Load setup (4.1)
20: Define saving directory (4.1)
21: Define file ending (4.1)
22: View original/processed image (4.4)
23: Select frame (4.4)
24: Zoom (4.4)
25: Start time-lapse (4.1)

Note: If the TLA is started in the basic user mode the elements 7-17, and 22 can not be seen as the image processing control panel is invisible. The area will instead be occupied by the preview panel(6), (see e.g. figure 4.2).

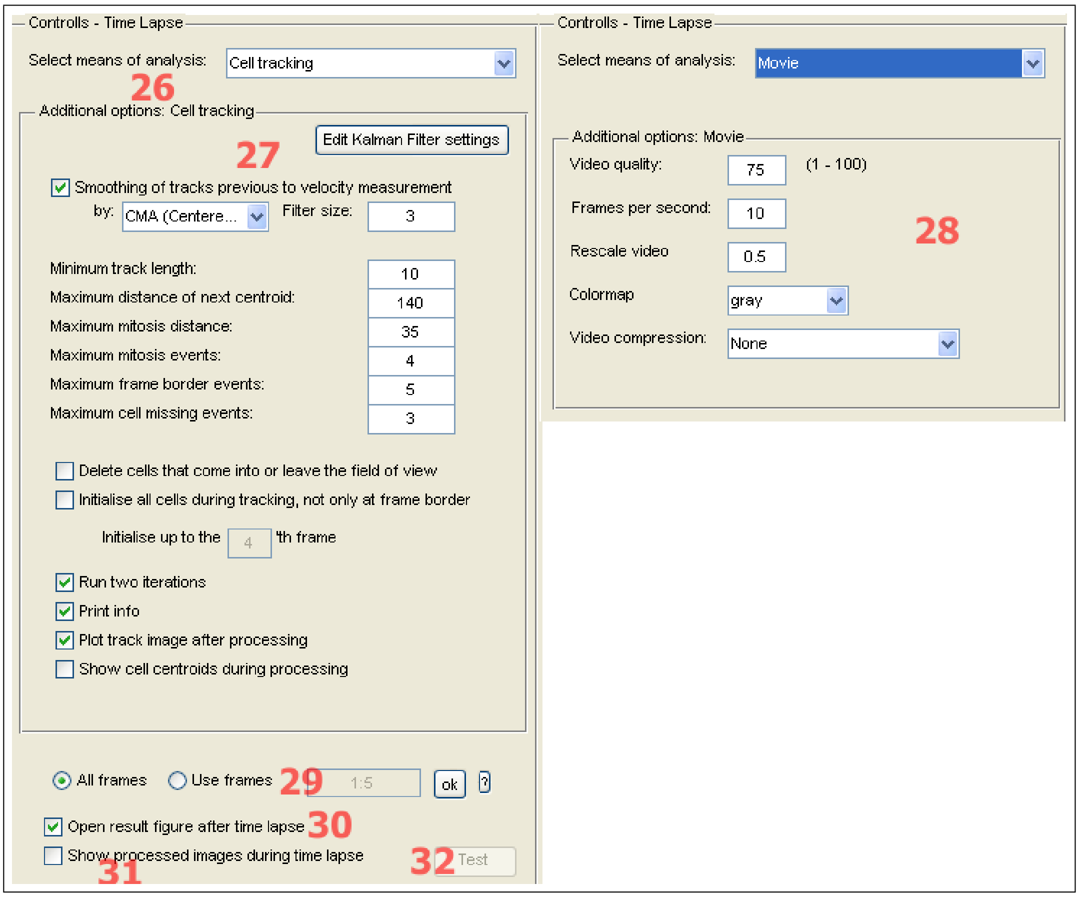

26: Select time-lapse analysis (8)
27: Cell tracking settings (8.2)
28: Movie settings (8.4)
29: Frame selection (8.1)
30: Open result figure after processing (5.3)
31: Show images during processing (8.5)
32: Test time-lapse (8.5)

3. Installing and running the TLA

The software is written in MatLab and the files are compiled as a stand-alone Graphical User Interface (GUI) program. This software requires either a MatLab version ($\geq$v. 7.2) or the MATLAB Compiler Runtime (MCR) bundled with the Time Lapse Analyser application. Download the current Time Lapse Analyser version. Unzip the Time Lapse Analyser package. Install the MCR by double-clicking it and following the instructions.

Windows:
Double click the tla.exe. The source file archive will be unpacked and the TLA will be started shortly after. Note, that if the TLA is started as a -stand alone application, the Matlab developer options will not be available. This information will also be displayed in the command window which is mandatory to keep the TLA running. If the command window is closed, the TLA will close without saving whatever file is processed at the moment. This is also the case if the TLA is closed using the window closing function of the window system.

Unix/Linux/MacOS X:
Call tla from the command line.

3.1. License

This work is licensed under a Creative Commons Attribution-Noncommercial 3.0 License.

3.2. Installation errors

If you receive an error during installing or running the MCR-Installer or the TLA it might be due to program version discrepancies. Make sure that the MCR-Installer has the same version number as the executable TLA file.

4. Basic user mode

The TLA was designed to enable fast and easy access for new users. The basic user mode requires only a minimum amount of user interaction and the handling is intuitive. This section provides the mandatory knowledge needed to use our program.

4.1. Running a time lapse analysis

After opening the TLA(3) only five steps are needed to start a predefined time-lapse analysis.

1. Load avi file / set of avis
2. Load a setup file
3. Define a saving directory
4. Define a name extension for the result files
5. Start the processing

The first step is to load the time lapse files. Do this using the Open avis / set of avis menu entry or the 'Open Avi' button (GUI map, item number 18).
The next step is to load a predefined TLA setup file (GUI map, item number 19). These files are constructed in the advanced user mode (section 6) and define the applied image processing operations

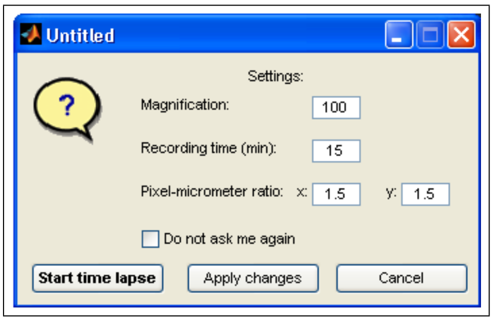

Figure 4.1: Dialogue to set the pixel size and the magnification.

and the sort of time lapse analysis. Example setups can be found on the projects homepage e.g. for multi-target-tracking on a set of cells.
Next, a saving directory and a file ending for the time lapse result (GUI map, item number 20,21) must be choosen. The results will automatically be saved under the avi file name which is extended by the user defined file ending. E.g. if the avi file list is Avi1.avi ··· Avi5.avi, and the result ending is defined as myresults, the results will be saved in the defined saving directory under Avi1_myresults.mat ··· Avi5_myresults.mat.
The last step is to start the time lapse processing (GUI map, item number 25). Once started, a 'Stop' button appears which can be used to interrupt the processing. This may especially be useful in the advanced user mode if some setup configurations must be adapted. If everything was done correctly, the dialogue displayed in figure 4.1 is shown. This dialogue will always shown previous to the time lapse analysis. If this is not required (i.e. if the settings never change) the dialogue can be switched of by checking the 'Do not ask me again' check-box. To undo this, use the menu entry Options→Advanced options→ Always check magnification.
If the settings are correct hit 'Start time lapse' and the analysis starts. For a detailed example see section 4.2.

4.2. Program sample

Here we describe a step-by-step example how to run a sample TLA setup. The example setup is used for multi-target cell tracking in DIC images. The mandatory cell confluence and image quality is shown by some example images which can be downloaded together with the setup file.

Load the avi example1.avi as well as the sample setup setup1.mat/setup1.zip from the projects homepage TLA homepage. Open the TLA and open the avi file (see the picture in figure 4.2).

Load the setup using the 'Load setup' button which will open a file browser window. Note that after this special setup is loaded, the programs appearance will not change, only the setups path and file is displayed in the Setup text field.

Next, select the menu entry Test image processing under Setup (see figure 4.3). It will take a couple of seconds to process the actual frame. The test function is used to check, whether a setup is suitable for the loaded avi or not. The loaded image processing is applied to every frame in the video example1.avi during the analysis.

After the image processing is finished select Options→Plot centroids (see figure 4.4). If this special setup is used on other cell images, it should be tested if all cells are detected (are marked by dots) and that the majority of cells is *not* multi-selected. This can be done using the centroid plot function

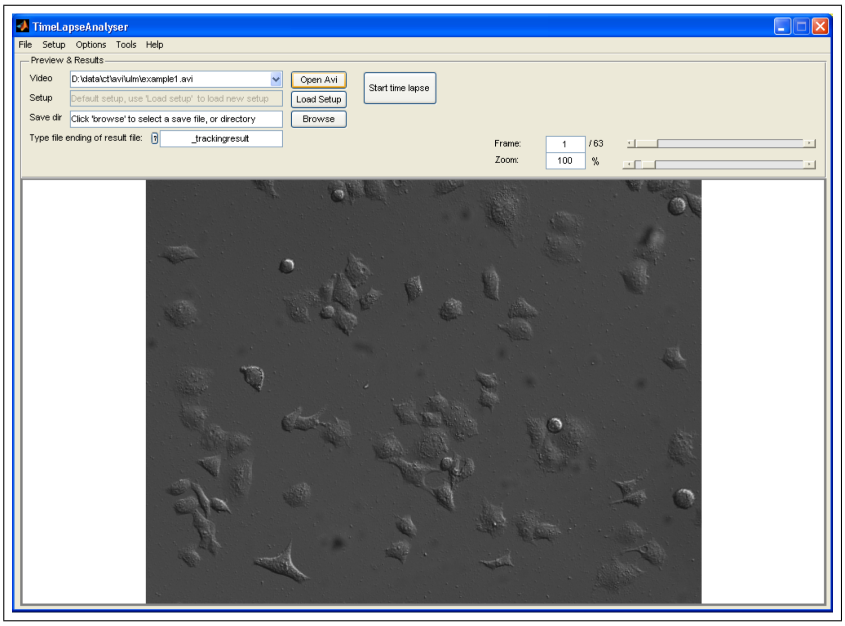

Figure 4.2: TLA start view after loading an avi file

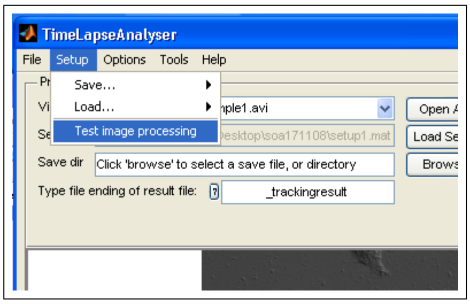

Figure 4.3: Test the image processing settings using the menu item Test image processing and wait for the TLA to compute the result.

(5.3)
Now either test some more images (scroll through the video and hit test image processing again) or start the time lapse analysis. Before the tracking process starts one has to define a valid saving directory and a result file ending. The default ending is "_trackingresult", the default saving directory is the folder "tlaresults" in the folder where the avis are located. After defining the saving path the pixel sizes, magnification and recording time must be confirmed. If the analysis is started, the Options→Plot centroids option should probably be unchecked again, otherwise the centroids are plotted anew for each processed frame, which will marginally slow down the process. However, one can leave it checked to validate if the centroids are found correctly in each frame. Each frame will take approximately five seconds to process (tested on an Intel Core 2 Duo machine with 2Gb RAM).

After the processing, a new program will open in which the tracking result can be evaluated. Also a cell track image similar to figure 4.5 is displayed. The result viewer program is further explained in section 9, an example for the viewer is displayed in fugure 4.6

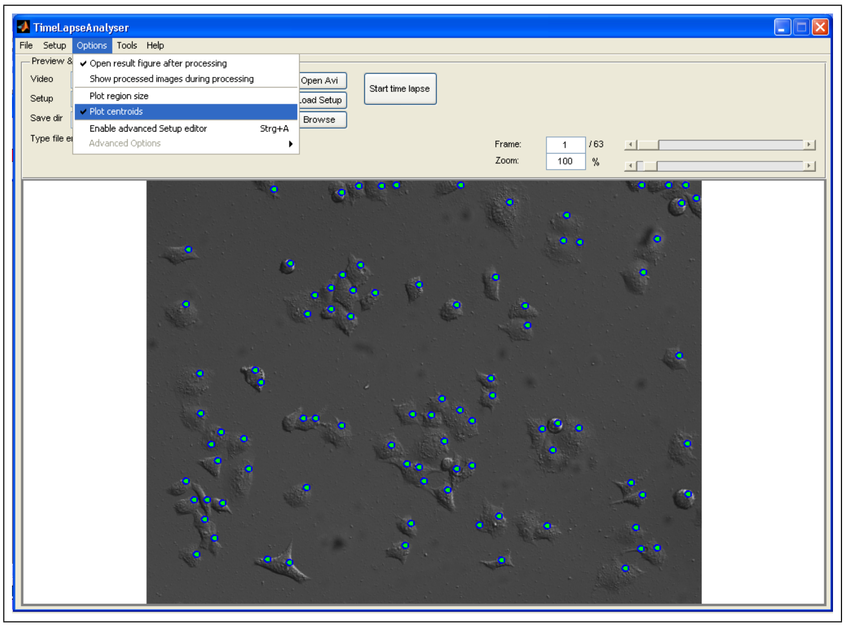

Figure 4.4: Validate the image processing on the loaded avi file before running an analysis. Using the first sample setup, each cell should contain a marked centroid. If to many cells are empty or contain far more than one centroid, the setup might not be suitable for the tested records. Centroids can only be plotted if the result of the image processing operation is a binary or label image.

There are some more example avis and setups on the projects webpage. In general the setups are provided together with some example images or videos and a readme.txt file for further important informations. Setup author are advised to read the appendix B where some conventions are introduced.

4.3. Useful functions

Test image processing

After a video file and a setup were loaded, it is possible to test the effect of the image processing settings on the avi frames via the menu entry Setup→Test image processing. The setup dependent image processing routine will be invoked and the result for the actual frame is displayed on the preview panel. The setup files (archives) should contain informations on how these result images should look like (either explained in the readme file or by sample images, see appendix B). If the setup does not work on the video files, one can either adept the setup or try another predefined setup file, if available.

Determine cell sizes

A possible source of error is the extend of the cells, as in most cases the image processing routines are adapted to cell sizes. The cell size and cell area can be measured using the menu entry Tools→Measure distance ···. Extrema for cell sizes should be mentioned in the setup readme file (if important), so applicants can check whether the setup is appropriate for their files or not. However, before the distance can be measured correctly, the settings regarding pixel-micrometer ratio have to be checked.

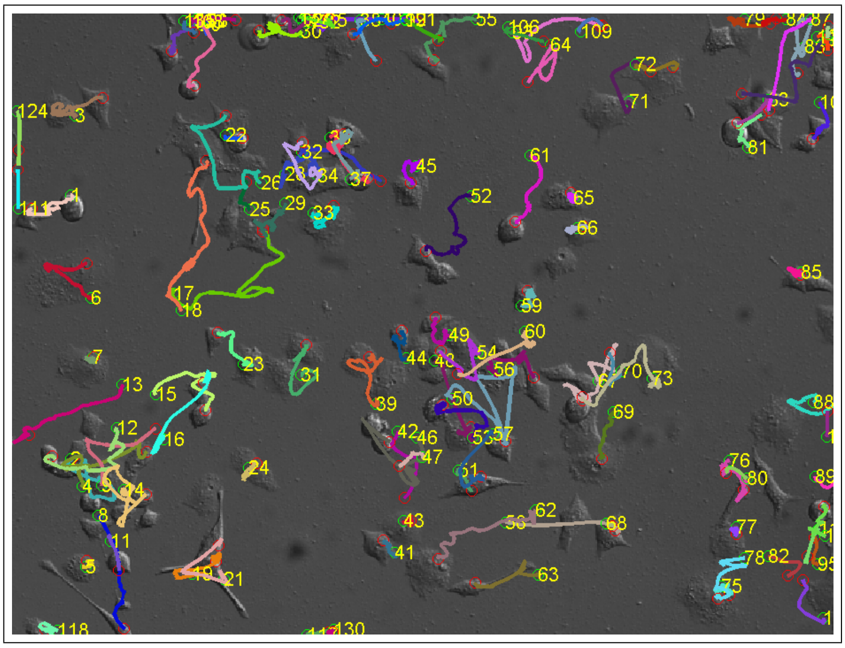

Figure 4.5: At the end of the time lapse analysis, the extracted cell tracks will be plotted and displayed.

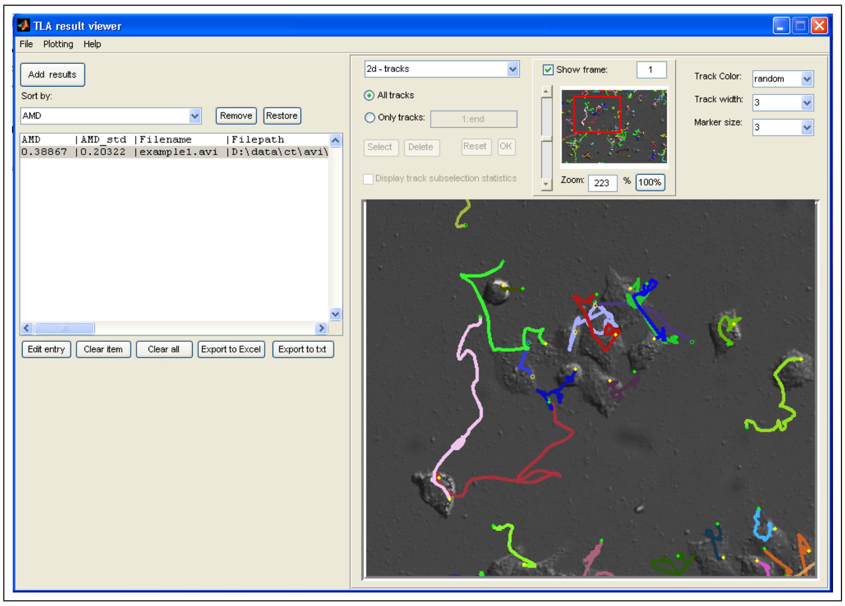

Figure 4.6: The TLA result-viewer is an extra program to manage results of multi target cell tracking.

Pixel-micrometer ratio

The used recording device and recording software should contain informations about the extend of one image pixel expressed in micrometer. This is important to measure image content and compute migration rates correctly. Sometimes, the x and y pixel extends are even different. The default settings ($x = 1.5, y = 1.5 \rightarrow$ one pixel has an extend of $1.5 \times 1.5\mu m$) can be changed under the menu entry Options→Change Pixel-extend, Magnification, Recording time. The settings Magnification and Recording time are important for cell tracking, to compute the average cell speed. As already said, these settings are also requested each time a time lapse experiment is started (see figure 4.1).

Scrolling through the video

If the result of the time lapse analysis does not seem to be correct, it may be due to single frames which were not recorded correctly or with low quality. To check this one can scroll through the video file using the scrollbar in the preview panel (Map item 23). Frames with exceptional appearance or low quality can be tested and so identified as possible error sources. If the results are strongly different from results of other frames (e.g. one previous, or the next frame), change into advanced mode and modify the setup, or omit the erroneous frames.

4.4. The preview panel

The preview panel is provided to verify image processing settings and to explore the video data. The user can scroll through a video sequence using the frame-scrollbar or by typing a particular image number into the input field. This can either be a number or the key-word "end" which denotes the last video frame. The maximum number of frames is displayed next to the scrollbar. Due to storage reasons, only the actual regarded frame is loaded, thus fast switching between images might not be possible if the images are large or the used machine is slow[1].

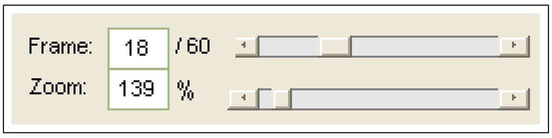

It is possible to zoom the image withe factors between 25% and 8000%. These are the minimum and maximum values of the scrollbar. If a lower/higher number is typed into the zoom edit field, one of the two extrema is set. A fast way to zoom back to 100% is to type some non numerical values into the edid field and hit return. The program will automatically switch back to 100%. However, the zoom factor will not change if the frame-scroll function is used or image processing is applied, nor will it switch back when a new avi is loaded.

If due to zoom settings the image content does not fit in the preview window, scrollbars are displayed to scroll through the frame, though it is also possible to move the focus by *dragging* the image in the desired direction. Left-click on the image, hold the mouse button down and drag the region of interest into the focus. The activation of the drag function will be indicated by a changed mouse button (change from single arrow to four-pointed arrow).

[1]The test images of size (1344×1024) are switched in real time on an Intel Core 2 Duo machine with 2Gb RAM

5. Menus

The following sections lists the menu options of the TLA. Many menu items can be *checked* and *unchecked* in most cases. A checked item is marked by a small *hook* sign ☑. Selecting the item again will uncheck it. Some menu options can be activated using short cuts. If available, they are named directly after the menu item name.

5.1. File

In this sub-menu images can be saved, videos and time-lapse-result files can be loaded.

- Open avis / set of avis [Ctrl+O]

  Open one or more avi files. The files are supposed to be either uncompressed or compressed with one of the following formats: Indeo 3, Indeo 5, Cinepak. Frames are converted to gray-scale images.

- Display Avi volume informations

  Display informations about the number of frames, frame size, video compression and further file informations in the command window.

- Change image and video settings

  Change some important settings for measuring image contents like the extend of cells. The extend of an image pixel in μm can be set. Also the magnification factor and the recording time of each frame can be set here. These settings are global, so if a time lapse analysis is applied on a stack of videos, it is assumed they were all recorded with equal settings (equal magnification, recording time pixel/μm ratio).

- Open image

  This menu item is used to open single images for processing. Time lapse processing is not possible if an image was loaded and the time-lapse panel will be disabled.

- Open result figure [Ctrl+F]

  Opens the RV with functions to display the results of the TLA if available. See section 9 for further informations.

- Save timelapse results as [Ctrl+S]

  If time-lapse results exist, this option will be enabled and gives the possibility to save the time lapse results. This is in general not necessary as all results will automatically be saved under a previously defined directory. The name is always defined by the avi file name and the user defined file ending (see 4.1 for more information).

- Load timelapse results

This function is only available in the advanced user mode and gives the possibility to load a time-lapse result file. While loading a result, the associated video file is first loaded if possible. If the file cannot be located by the system, the user will be asked to locate the file again.
Each result file contains the TLA configuration settings that were used to produce the result. The user must decide if this configuration should be loaded, as well.

A TLA result of a cell tracking experiment contains the cell centroid position of each detected cell for every frame. After loading such a result, one can run the tracking again without the need for previous image processing. This makes it possibile to adjust some tracking settings and test the effects immediately.

- Clear images & avis' [Ctrl+C]

  Clears all loaded images and video files. If the program runs extremely low or a time lapse experiment was interrupted , a cleanup may help.

- Reload [Ctrl+R]

  Reloads the program. Restores the default settings. The user will be asked first if he/she likes to store the actual configuration in a setup file.

- Exit (Save configuration for next start) [Ctrl+X]

  If the TLA is closed using this menu entry or its shortcut [Ctrl+X] the actual configuration is stored and automatically restored when the TLA is started the next time. This does not effect any file settings like the avi file list or the saving information, but basically all other storable informations will be saved and restored.

- Exit [Ctrl+Q]

  Closes the program without saving the current configuration.

5.2. Setup

Here configurations (setups) can be saved and loaded. Setups are stored configurations of the TLA and define the image processing on the single frames as well as the sort of time lapse analysis.

- Save [Ctrl+S]

  Save a TLA setup file. This can either be an entire setup (→Entire setup), only the image processing panel settings (→image processing options) or only the time-lapse panel settings (→time lapse options).

- Load [Ctrl+L]

Use the menu item Entire setup to load a previously defined setup file. If only image processing or time-lapse settings should be loaded, use image processing options or time lapse options instead. One can also load entire setups using the button 'Load setup'(Map item 19) on the preview panel.

- Test image processing

  After loading a video file (or files) and a predefined setup testing the image processing on the data first and -if provided- comparing the result to additional sample images may be a good idea. By hitting Test image processing the actual displayed image will be processed. If the results is sufficient one can start the time lapse analysis. If the result strongly differ from what is expected, the workstack (7) can either be modified using this manual, or the setup author should be ask for help. Just running the time lapse analysis will in this case most probably return unreliable results.

5.3. Options

- ☑Open result figure after processing

  If this item is checked, a new program is automatically launched after the time-lapse analysis is finished. Which program is opened is in principle dependent on the kind of selected time-lapse analysis. At present, only for the cell tracking analysis a result viewer is available. The RV is explained in chapter 9.

- ☑Show processed images during processing

  If this menu item is checked each processed frame is displayed in the preview panel during the cause of the time-lapse analysis. This, however, means that the TLA's graphical interface is constantly updated during the processing which may slow down the computation. This option is thus useful to test if the image processing works for the first few images.

- Plot region size

  If the result of the automated image processing routine is a binary or segmented image, the sizes of each distinct region is plotted if this option is selected. It is likely that a majority of the setups will contain image processing routines which use certain region sizes as thresholds (e.g. to delete small non cell tissue regions in an segmented image). If the image processing functions are tested, and the results are not comparable to provided reference image this option may help to find an easy solution or provide at least some hints.

- ☑Plot centroids

  Use this option to mark the geometrical centroids of segmented regions in the actual processed image. If the image is not a binary image or does not contain any centroids (see workstack `'Centroid'` function, see section A.3), this option has no effects. If the advanced user mode is activated and one switches between the original and the processed image, centroids are plotted in either one if available.

- Display number of regions

  If the result of the image processing operation is a binary image this menu item is used to display the number of independent regions in the image. If the result is not a binary image this function is disabled. Note that even if an image might look pretty black and white (two-coloured, thus binary) it might not be a binary image. Check the number of used gray values with the menu item Show image information and histogram.

- Enable advanced setup options [Ctrl+A]

  Switch between basic and advanced user mode.

- ☑Advanced options

  If the workstack contains a high number of entries, the ☑Display workstack informations menu item h elps to get a better overview. While running the image processing routines the workstack functions are displayed in a structured and more intuitive way in the command window (see e.g. example figure 7.2)

  If external MatLab implemented image processing functions should be used one can select the ☑Enable MatLab developer options menu item. This enables the option to test new functions on the image processing panel. For more informations see section 10.

5.4. Tools

- Print screen [Ctrl+P]

  Makes a screen-shot of the TLA.

- Show image information and histogram [Ctrl+I]

  Plots the gray value histogram of the actual displayed image in a separate figure window if this is possible. If a merged image (see 6.2) is currently displayed, the histogram of the original image will be plotted. Additional information like the image size, as well as the minimum and maximum used gray value are displayed in the figures title.

- Extract frame/image

  Opens the actual displayed image in a separate figure window. This function is used to save an individual image. The standard MatLab figure window allows to store the image in many different formats like *.png, *.pdf etc..

- Measure distance in micrometer [Ctrl+D]

Activates the distance measurement tool. It enables the user to select a number of points in the frame using the left mouse button. The cumulative distance between the single points is computed and displayed in the image after the point selection is finished. The selection ends if either the right mouse button or enter are hit. At least two points have to be selected with the left mouse button. The point clicked with the right mouse button is not included in the point selection. During point selection one can also delete points in reverse order by hitting the delete or backspace key. Before making a measurement one should check if the pixel-micrometer ratio is correctly set under the menu item Change image and video settingsin the filemenu(5.1). For possible measurements see example figure 5.1.

- Measure area in micrometer

  Activates the area measurement tool. The area in the manually defined polygon, is returned (unit is μm, three selected points are at least required). The polygon is automatically closed by connecting subsequent selected points as well as the first and the last point (see figure 5.1).

- Measure distance in pixels [Ctrl+E]

  Similar to measurement of distance in micrometer. Only the returned number has *pixels as unit.* This measurement is independent from the pixel-micrometer ratio (see figure 5.1).

- Measure area in pixels[Ctrl+Y]

  Similar to area measurement in μm. Unit is pixels (see figure 5.1).

- ☑Show pixel intensity [Ctrl+G]

  If this menu item is checked the image gray value under the mouse pointer is displayed in the preview panel. This might be helpful to set some thresholds in an image processing setup (see figure 5.1).

- Image line profile [Ctrl+W]

  Select a polygon for which a line profile is plotted in a separate 2d plot. A line profile is the gray value intensity of all pixels under the selected polygon. (See figure 5.2).

- Change colormap [Ctrl+M]

  Opens a dialogue where different *colormaps* can be selected. Colormaps are used to pseudo colour actual frame. This will only influence the appearance of the image, not the image content itself. (Note: The recently used colormap will also be used if a new video file is written, see sections 8.4 and 9.3)

- ☑Show colorbar

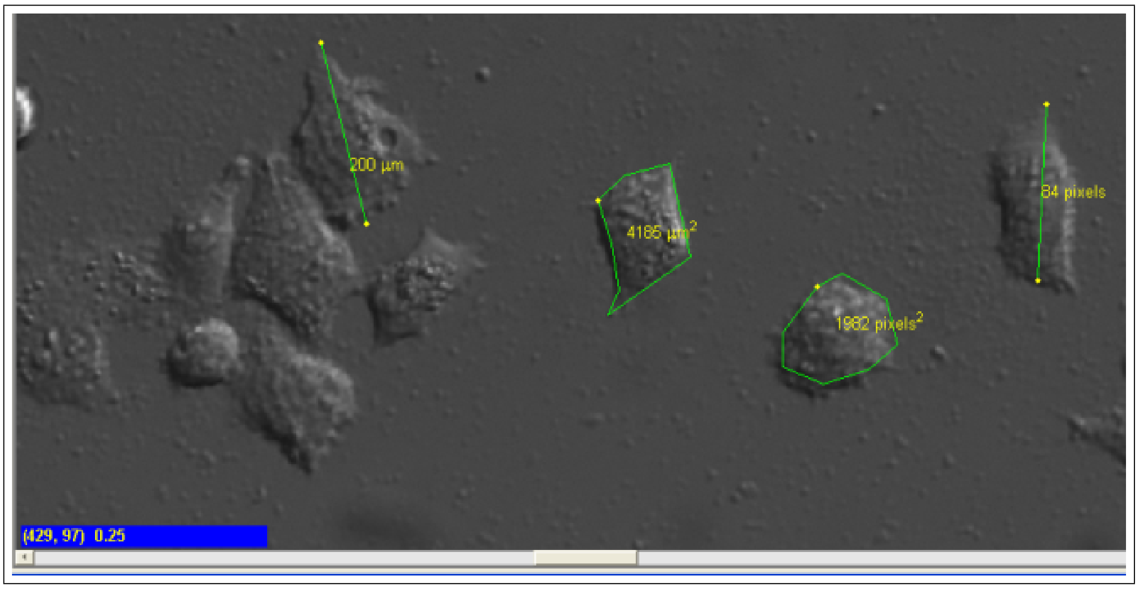

Figure 5.1: Examples for the measurement functions of the TLA. One can measure distances and areas in μm and pixel. Using Show pixel intensities the actual pixel intensity under the mouse pointer is displayed in the lower left of the preview panel (blue box), together with the pixels (x,y)-coordinates.

Displays a colorbar right beside the image. Colorbars show the used colours of the colormap and the associated pixel intensities.

5.5. Help

- Help [Ctrl+H]

  This will open the projects website where quick introductions and setups can be loaded. Also new program versions will be provided on this page.

- About

  Displays program version informations.

6. Advanced setup construction mode

The next sections will provide the informations needed to build own image processing and segmentation routines. In general, when the advanced mode is enabled an *image processing panel* and a *time lapse panel* will be opened. A central element for the image processing is the *workstack* which is in detail explained in section 7. Basic image processing functions are combined in this stack to more powerful

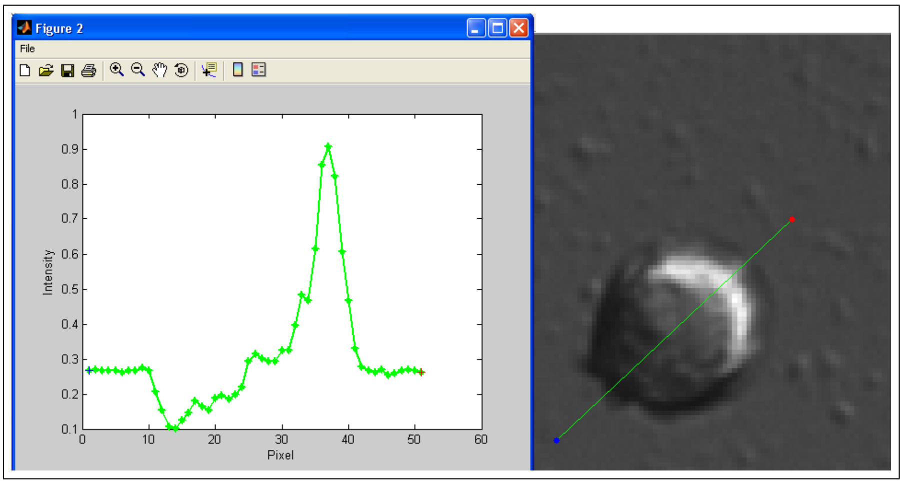

Figure 5.2: Example of an image line profile. The gray values along the green line (from the blue to the red dot) are plotted into a separate figure.

functions. A combination (or *workstack entry*) can be saved in a quick option list (section 6.1) or in a file for later use. The available image processing functions can be found in appendix A. The different time lapse options so far provided are introduced in the appendix.

6.1. Quickoptions

The *quickoptions* provide an easy way to manage often used image processing functions, or better, often used *workstack* entries. The quicklist consists of a popdown list which basically includes some predefined image processing routines. If a list item is selected, the associated `'entry'` is displayed in the WS.

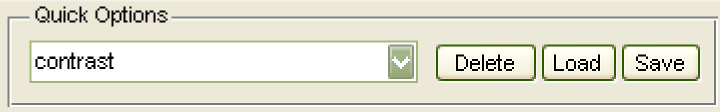

The list options can be extended using the '2QO' button (see section 7.7). Quick option lists can be loaded and stored using 'Load' and 'Save', and most of the list entries can be deleted using the 'delete' button (some default items cannot be deleted). Before deleting entries, an actual version of the list should be saved as backup.

6.2. Test the image processing

Image processing routines can be tested on the actual displayed image using either the menu entry Setup→Test image processing or the 'run'-button on the image processing panel. If the WS-entry is formally correct, the image processing routines are invoked and the resulting image is displayed in the image window. One can then switch back and forth between the processed and the original image using the 'Orginal' and 'After Processing' buttons.

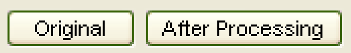

If the result of the image processing operations is a binary image (pixel are either one or zero), by default an overlay image is displayed. This however is only for convenience and evaluation of the

result. The result itself will not be changed. If the automatic image merging is not required, it can be switched of using the checkbox displayed in the figure below. The intensity of the superimposed binary image can be adjusted using the slider next to the checkbox where 0 corresponds to no overlay at all, and 1 to the most intense overlay. As already mentioned, regional centroids of image regions

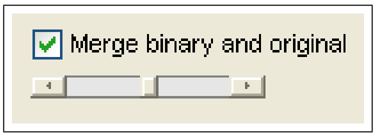

in binary images can be displayed as well (see section 5.3).

7. Workstack - defining image processing work flow

This section has the purpose of introducing the interested reader to a central element in the graphical user interface (GUI) of the TLA: the *workstack*(WS).

The WS contains all operations that are imposed on the frames and provides broad possibilities to modify image content. At first, some basic ideas regarding the structure of information processing are introduced and afterwards we provide some interesting applications.

7.1. Expressions, Units, Operations

The WS holds so called expressions which are explicitly or implicitly grouped in triplets. Expressions always result in images. The first element of an expression can be a *unit* or an expression, the second element must always be a basic *operation* (namely `'+,-,/,*,usefor`[2]`'`) and the third element can again be a unit or, if the `'usefor'`-operation is not used, an expression itself. A single unit is also called expression as it returns an image as result.

A unit is for example the median operation on an image `'median(mask)'`. Units always *are*, or *return* an image. Another way of seeing units is as more or less complicated image processing functions. The most basic function is `'Original'` which is the identity function and returns the original image itself.

The name *workstack* was chosen, as in the simplest case, the expression in the WS is processed iteratively from the first to the last unit. The following example 1 will illustrate this with a simple expression:

Example 1:

```
1: Original
2: -
3: Blurring(100)
4: *
5: 0.2
```

In example 1 the units are `'Original'`, and `'Blurring(100)'`. As already said, `'Original'` returns the input image, and `'Blurring(100)'` is a version of the original image, filtered with a square shaped averaging filter mask of size 100×100. The basic operations used are `'-'` and `'*'`. Basic operators

[2]see section 7.3

work always pixel per pixel, which let the `'0.2'` at the end of the stack be interpreted as an image which has the value 0.2 at every pixel position (image values always range from 0 to 1). The processing is here as follows: "Take the original image, subtract the blurred original image and afterwards multiply pixel per pixel by 0.2". The entire example is an expression.

The next example 2 shows an invalid workstack entry as only one unit is used with one basic operator:

Example 2:

```
1: -
2: Original    \\WRONG!
```

The intended meaning of this entry is obviously to produce the inverted original image. To achieve this with a valid expression simply use the `'Invert'` operation.

7.2. General process flow

Regarding the general process flow, there is no hierarchy given for the operators `'+,-,*,/,usefor'`. The structure that defines which units are processed at first is given by the position in the stack. In the first example these are the first three elements (`'Original - Blurring'`) . The result is then used in the consecutive operations, that is, the result of (`'Original - Blurring'`) is multiplied by 2.

The only way, and also the only necessary element to structure the stack are *brackets* which can be used explicitly, but which are also used implicitly as the stack is always organised in tripplets. The WS-entry of the first example 1 will have the implicit representation

Example 3:

```
1: (
2: Original
3: -
4: Blurring(100)
5: )
6: *
7: 2
```

The expressions from example 1 and example 3 are identical, whereas the following example 4 has a different process flow

Example 4:

```
1: Original
2: -
3: (
4: Blurring(100)
5: *
6: 2
7: )
```

In this case, the expression in brackets is subtracted from the original frame. Thus the expression (`'Blurring * 2'`) must be processed first and the work flow is directed by the explicit brackets. The general work flow in the workstack can be deduced from this example 4: Expressions in brackets are always processed first. If no explicit bracketing is given, implicit bracketing is used and the expression is processed from the top to the bottom.

7.3. Usefor - a basic operator

Beside the most basic operations `'(+,-,*,/)'` the `'usefor'` operator is necessarily provided. The next example should intuitively highlight the meaning. Here a (5×5) median filter is used on the original image. Then the result is used for the "adjust" operation.

Example 5:

```
1: Original
2: usefor
3: Median(5)
4: usefor
5: Adjust
```

The `'usefor'` operator must be used whenever one of the image processing functions (units) should be used on already processed images. In the expression `'A usefor B'` the expression `'A'` must result in a valid image and `'B'` must be an operation that can be used on the result of `'A'`. Note that `'B'` cannot be another expression. As a simplification, the `'usefor'` operator is also used implicitly, when two expressions are subsequently placed without a basic operation in between. The appearance is then again more "stack like" and intuitive:

Example 6:

```
1: Original
2: Median(5)
3: Adjust
```

At this point it is useful to highlight the bi fold functionality of the units. In a certain manner units are overloaded as they can be used as both, single stand alone units, or as a function that can be applied on previously processed images.

The unit `'Median(5)'` returns the median filtered original image which is equivalent to the expression `'Original usefor Median(5)'`. A unit is stand-alone if its previous entry is not the usefor operator. In this case, the original image is always taken for the denoted operation:

`'[...]  + Blurring(50)'`
equals
`'[...]  + (Original usefor Blurring(50))'`

whereas in
`'A usefor Blurring(50)'`
the blurring operation is applied on the result of expression `'A'`.

Usefor - Wrong application

From the behaviour of the units as functions the correct application of the `'usefor'` operator can be deduced. After the `'usefor'`-operation, expressions with more than one units are not allowed (or are not interpreted in the intended way). The mandatory entry after `'usefor'` is a single unit that works as an image processing function. In the next (bad) example 7, the subsequent expression after the usefor operation is not a single unit but the expression `'(Original-Blurring(50))'`. This makes no sense, as the expression is no function to be applied on the result of the `'Adjust'` operation.

Example 7:

```
Adjust
usefor
(         \\WRONG
Original
-
Blurring(50)
)
```

As the bracketing in this case makes no sense, it will not be regarded by the computer. The implicit representation will thus be
`'Adjust usefor Original - Blurring(50)'`
which is of course (because of the work flow, and because `'Original'` is the identity function)
`'Adjust - Blurring(50)'`.
If we go one step further and insert the implicit representation of the stand alone units `'Adjust'` and `'Blurring(50)'` we get the final stack as it will be processed by the computer:

Example 8:

```
(
Original
usefor
Adjust
)
-
(
Original
usefor
Blurring(50)
)
```

This example only displays the implicit representation of a workstack entry. For convenience, only the most compact representation is used in the following sections (here: `'Adjust - Blurring(50)'`).

7.4. Complexity -he `'#'`-perator

The power of the so far introduced WS functions in combination with the bracket-ependent work flow is not sufficient to work efficiently in many applications. Imagine one wants to use the result of a complicated and time consuming image processing operation twice or even more times (e.g `'Blurring(mask)'` can be slow for `'mask'` high). The possibility up to now would be to do the operation as often as the result is needed, which leads to unreasonable time consuming image processing.

Other problems arise in *time lapse imaging*, when results from previous images should be used in the workstack. This is not possible with the current introduced system.

To account for these necessary functionality, the #-perator (hash operator) is introduced. The #-perator is a reference on already processed parts of the workstack, either applied on the actual frame, or on previous frames if time lapse imaging is used. A small example will enlighten the meaning:

Example 9:

```
1: Original
2: -
3: Blurring(200)
4: *
5: \#([1,3],0,1)
```

The #-operator always requires three arguments. The first is the interval of the referenced workstack part, in this case, the interval is defined to range from the first to the third line, thus includes the operations (`'Original - Blurring(200)'`). The second argument passes the information, which frame is referenced. The reference is always denoted in relation to the actual frame. As the reference can only point toward already processed frames, the reference is either negative (e.g. minus one for the frame previous to the actual frame) or zero, if the result on the actual frame should be used as is the case in the example. The last argument denotes the alternative that should be used if the referenced result is not available yet. This can be the case, if the result from a previous image in a time-lapse experiment is referenced, but the processing has just started (see example 14).

Given these informations, it becomes clear that example 9 returns the same result as

Example 10:

```
1:  (
2:  Original
3:  -
4:  Blurring(200)
5:  )
6:  *
7:  (
8:  Original
9:  -
10: Blurring(200)
11: )
```

but only in half the time as the expression `'Original - Blurring(200)'` is processed just once. For the sake of completeness we name here the operator `'Power(2)'` which can also be used to produce the result from expression 9 and 10:

Example 11:

```
1: Original
2: -
3: Blurring(200)  // Mind the process flow! First the subtraction...
4: Power(2)
```

7.5. Convenient use of the workstack - compression of lines

As the number of lines in the workstack is growing fast in more complicated applications the line compression is introduced as a possibility to ”pack” closed expressions. The inner representation of the WS stays the same, only the outer appearance is changed to a simpler form. Imagine the following (still simple) WS entry

Example 12:

```
1:  (
2:  Original
3:  -
4:  Blurring(200)
5:  )
6:  Median(5)
7:  Adjust
8:  Otsu
9:  Opening
10: Delete small regions(500)
11: Centroid
```

This looks more complicated than necessary. The process flow is here: ”Take the difference image (`'Original-Blurring(200)'`), apply a median filter on the result, adjust the grey value of the result. Then binarise the image using Otsu thresholding, apply morphological opening on the result, then delete all segment regions which are smaller than 500 pixels and finally return only the regional centroids.”

It is obvious, that the first part here is the image preprocessing and the second part the image binarisation. Therefore the stack can e.g. be compressed to

Example 13:

```
[1-7]:  IPP      // Compression of the first seven lines
8:      usefor
9:      Otsu
10:     Opening
11:     Delete small regions(500)
12:     Centroid
```

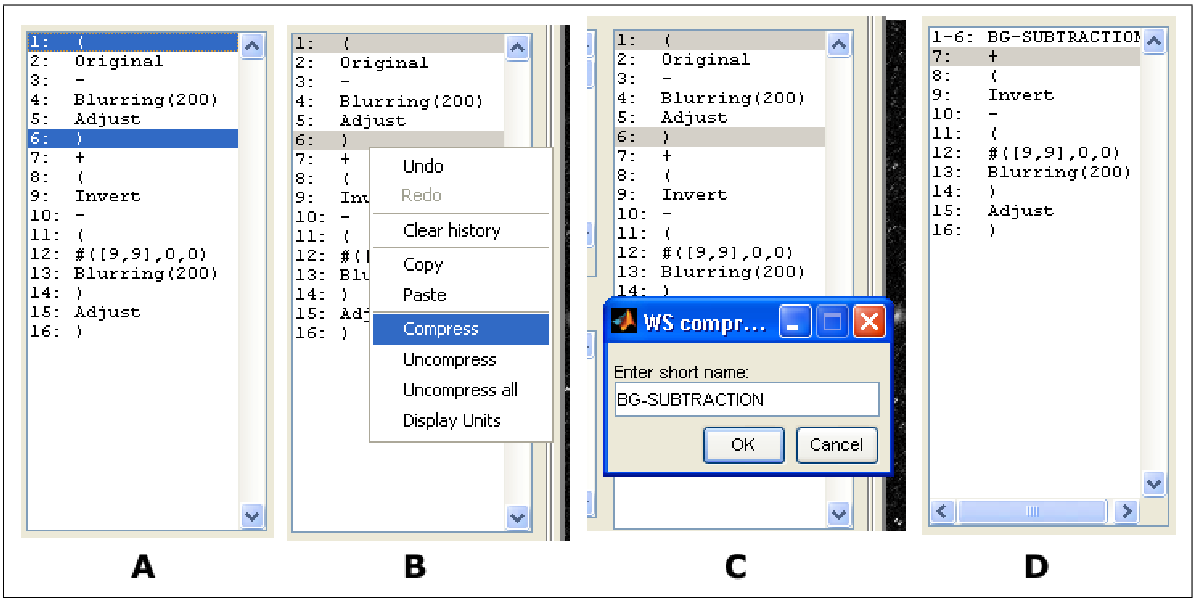

Figure 7.1: Illustration of line compression. If an opening bracket is clicked on, the corresponding closing bracket is also marked. Right click on the workstack in image A will open the context menu. Select the Compress sub-menu item. A dialogue box will open where a short name for the compressed stack entry has to be entered. Confirm the dialogue and the lines will be compressed using the defined name. The number of compressed lines is written in front of the compression name for reference reasons.

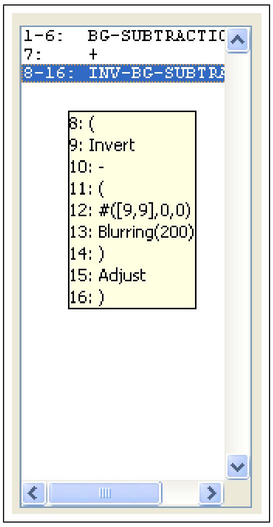

The first seven lines where compressed to the module IPP (for a better handling, the compression name should be placed in upper letters). The interval of compressed lines is always written in front of the selected module name for a possible reference by the '#'-operator. Compression names *must* not be unique, e.g. two different image processing expressions can be assigned the same module name, but for the sake of simplicity and the better usability, unique names *should* be used. Also for convenience trivial suggestions for names are made by the system. It is of course as easily possible to *unfold* the compressed lines to modify certain lines of the interval. As another help toward easy compression of lines, only opening or closing brackets of an expression have to be selected, then the *compression* function in the context menu called and the entire expression in between the selected bracket and its counterpart bracket is compressed. A graphical illustration of line compression is shown in figure 7.1.

A final note: if a compressed list is highlighted (marked) and the cursor still is hold over the workstack a tool tip string will pop up, displaying the entire compressed list (See figure 7.5).

7.6. WS examples

In the next section some interesting WS-entries contained in the quick option list 6.1 are introduced as well as some additional ideas just to provide better insights into the functionality of the #-operator. All used filter sizes are adjusted to a frame size of (1024 × 1344) and a cell size of about 30 to 80 pixels.

7.6.1. Mean stack image

Assuming one wants to compute the mean image from an image stack containing 50 frames. The general approach is to divide each single frame by the total number of used frames, and add consecutively add up the results. Example 14 translates this idea into a workstack entry

Example 14:

```
1:  Original
2:  /
3:  Number of frames
4:  +
5:  #([1,5],-1,0)
```

Starting with image one in the first iteration, the referenced image in line number five is still empty as the image zero is not existing and could therefore not be processed. Hence the result in the first iteration is the first frame divided by the number of frames plus the alternative image which has the value zero at every pixel. In the second iteration the second frame is divided by the total number of frames and is added to the result from the previous iteration etc.. Note, that if the #-operator would refer to the actual frame (`'#([1,5],0,0)'`), the reference could never be evaluated and the result would only be the actual frame divided by the total number of frames.

7.6.2. Cell tissue filter

Purpose of the next WS-entry is to separate cell tissue from background of an image without dividing individual cells.

Example 15:

```
1:  Scale(.5)
2:  Entropy(9)
3:  Median(25)
4:  Otsu
5:  Delete Small Regions(200)
6:  Scale(org)
```

First of all, the image is scaled to a quarter of its size (each side is scaled to half its size). This is to speed up the more time consuming `'Entropy(9)'` operation. The local image entropy is used here to highlight structural information in the image. The following median filter reduces noise before the Otsu-threshold is computed to binarise the image. Then cell regions to small to be living cells are deleted and finally the image is rescaled to its original size.

7.7. Workstack buttons and context menues

In general workstack items are selected by double clicking on them. An option dialogue will open where parameter of the selected function can be adjusted. Confirming the dialogue will insert the

selected function in the workstack. In general, items are always added at the actual marked position in the workstack. The marked item and all lower items will be pushed down. Only if the last workstack element is marked, the selected function will be inserted at the end.

+ Addition

Adds a "plus" sign at the actual marked position in the WS. This operation denotes pixel-wise addition of images.

- Subtraction

Adds a "subtraction" sign at the actual marked position in the WS. This operation denotes pixel-wise subtraction of images.

* Multiplication

Adds a "multiplication" sign at the actual marked position in the WS. This operation denotes pixel-wise multiplication of images.

/ Division

Adds a "division" sign at the actual marked position in the WS. This operation denotes pixel-wise division of images.

( ) Brackets

Adds an opening/closing bracket at the actual position in the WS.

usefor Usefor

Adds the usefor operation to the WS.

up Up

Moves the actual marked WS-item or items one position up if that is possible. This can also be done with the left arrow button on the keyboard.

dn Down

Moves the actual marked WS-item or items one position down if that is possible. This can also be done with the right arrow button on the keyboard.

#-operator

Inserts the #-operator in the WS.

<< 1 Insert number

Inserts the actual number displayed in the edit-field right beside the button into the WS.

Delete

Deletes the actual marked item or items. Can also be achieved using the delete button on the keyboard. If the backspace key is used, only the item *before* the selection is deleted.

Delete all

Replaces all items by the item `'Original'`.

Change

If the actual marked item requires additional parameter, the parameter dialogue box is again opened so that some adjustments can be made.

2QO

If the actual WS-entry is it worth to be transfered into the quickopion list use this button. A dialogue box will open where a short name for the entry has to be defined. After confirming the dialogue, the WS-entry is stored in the actual quickoptions list. Note that the name *must* not be unique, but it should be for convenience.

Load

Load a previously saved WS-entry.

Save

Save a WS-entry. Use this option to save the WS-entry.

7.7.1. Context menu

The context menu of the WS holds items to *undo* or *redo* the last actions applied to the WS. *Clear history* will delete the entire action history. Furthermore the context menu holds the functions *Compress*, *uncompress*, *uncompress all* which are explained in section 7.5. The function *Display Units* is used to display the WS list in a more structured way in the command window (see figure 7.2). Here the interleaving of the WS-commands is - sketched by indentions and all brackets - implicitly and explicitly are displayed.

8. Time lapse functions

Beside the various possibilities of the image processing settings, the time lapse functions of the TLA are also adjustable in certain ways.

At first one can select the kind of analysis which is in the current version (v-0.1.2) either cell tracking, video writing or just the stack image processing. This is done via the pop down menu on the time lapse panel. Special adjustments for the distinct analysis are introduced in the following sections.

However, there are some settings which are common for all time lapse function. One is the frame selection, which is introduced in the next section 8.1. Then in general, two other settings are available.

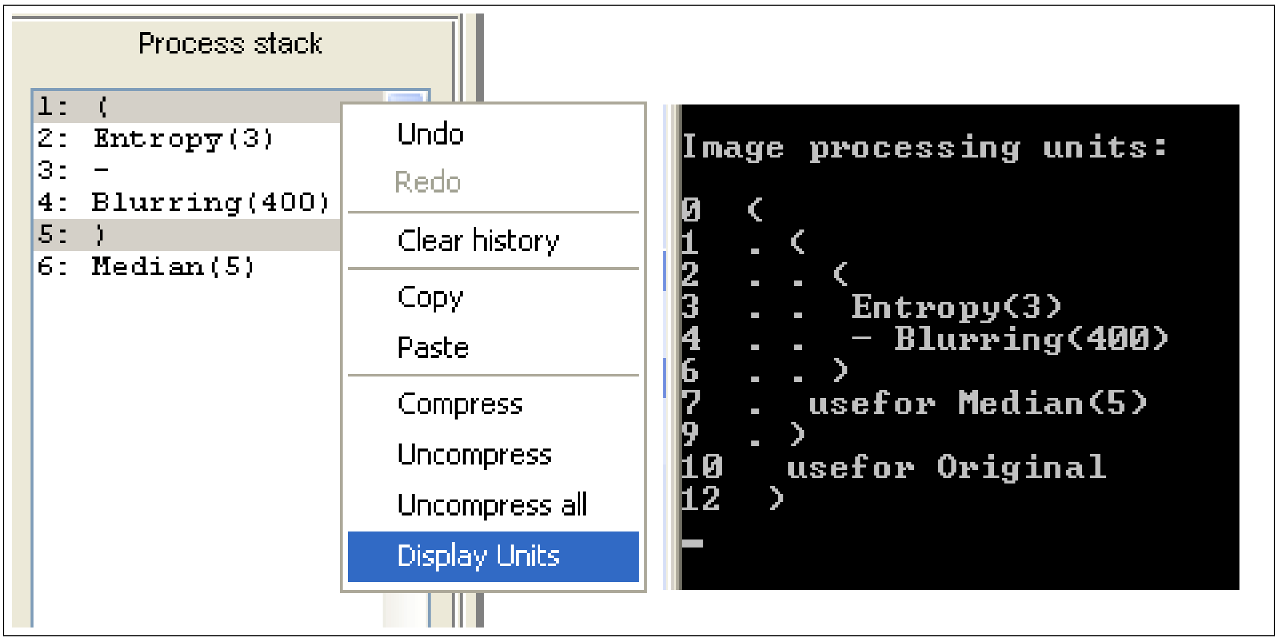

Figure 7.2: Context menu of the workstack

One can decide if the processed images should be shown during the time-lapse analysis via a check box at the bottom of the time-lapse panel or via the menu entry Options →Show processed images during processing. If this is the case, the preview panel always displays the last processed image. If selected and appropriate, regional centroids are also plotted into the processed images (see menu item Options 5.3). This will however slow down the processing as the graphical user interface (GUI) has to be updated for each new frame.

The second general setting enables/disables the opening of a result figure after the time-lapse processing ended. If the check box or the menu item Options→Open result figure after processing are selected, a result viewer program will be launched after the analysis, if available. So far, this concerns only the life cell tracking. The associated result viewer is explained in detail in section 9.

8.1. Frame selection

On the time-lapse panel the *frame selection edit box* provides the possibility to define the list of frames that will be used in the time-lapse experiment. Either one takes all, or a sublist of frames. Hereby one can make use of the *colon operator*(:) and the key word *end* (lower or upper letter does not matter). Using the colon operator is an easy way to write down monotonous increasing frame lists with little effort. To produce a list, one defines a starting point, a step width and an end point. The entry 1:2:10 will e.g. define the frame list [1 3 5 7 9]. It starts at one, increases in steps of two and ends at a maximum of nine. Note that the ten is not included as it does not fit into the scheme of increasing numbers. If the step number is omitted, the step size of one is assumed. (1:5→[1 2 3 4 5]). If instead of the last number the word *end* is used, the last frame from the actual processed video file is used (thus the maximum possible frame number). The list entry can be checked by hitting the 'ok'-button right beside the edit field. The defined list will be displayed in the command window.

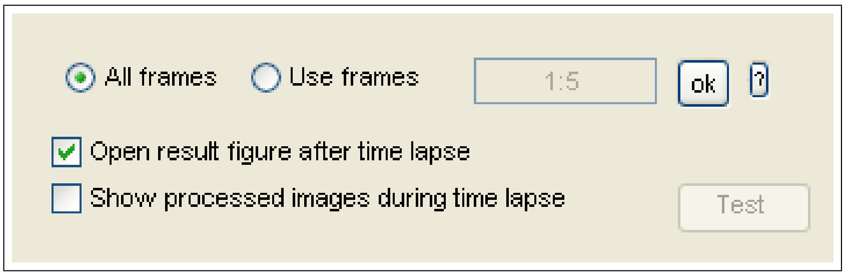

Figure 8.1: Frame selection and additional displaying functions for a time-lapse analysis

8.2. Cell tracking - multi target tracking

Automated multi target cell tracking was the initial reason to implement the TLA. Cell tracking is increasingly gaining importance in high throughput applications and of vast interest for the life science society. Although our system aimed at tracking life cells *in vitro* the therein included demands for robustness and adjust ability regarding the various different cell types and imaging techniques may also be advantageous for other areas where data association tracking is needed. If in general the problem is to track a high number of targets which can be approximated sufficiently by their centroids, our system may be of assistance.

In this first version we included a point based tracking method consisting of a Kalman Filter (KF) for motion modelling and a higher level monitoring module[3]. The settings of the KF as well as the monitoring module (MM) setting are further explained in this sections.

Kalman filter settings

Hitting the 'Edit Kalman Filter settings' button will open a dialog where the movement model matrix of the KF, the matrix which defines the relation between state and measurement, the measurement error covariance matrix and the system error covariance matrix can be adjusted. The basic settings are a constant movement and standard normal distributed noise. Also, the measurement will be directly translated into the new state without any change. The fixed design will be improved in future versions of the TLA.
The next settings relate mostly to the implemented higher level decision module or monitoring module (MM) as we call it.

Track smoothing

Choose a smoothing filter for the resulting cell track. This will reduce noise and stabilize the tracking result. The choice is between a centered moving average filter with free adjustable window size and a cubic-spline smoothing filter.

Minimum track length

All tracks consisting of less than the defined minimum number of centroids are deleted after the analysis. E.g. if the minimum is 10, only tracks with 10 or more centroids are used for the final AMD computation.

[3]Read more about the implemented tracking system in the paper *A fully automated tracking system to significantly improve the precision of cell migration analysis in time-lapse video microscopy* and the associated supplementary material. The paper is located on the same web page as the TLA-programm.

Maximum centroid distance

This distance defines the maximum possible displacement of a cell from one frame to the next. It is better to be rather generous with this setting. The distance is given in pixels and can be estimated using the distance measurement tool described in section 5.4.

Maximum mitosis distance

This threshold defines the distance of two individual cells directly after the mitosis division process. If two cells are closer to each other than the defined maximum mitosis distance, they are marked for a possible mitosis event.

Maximum mitosis events

If centroids are marked several times for a possible mitosis event the cells are regarded as merged. The maximum number of mitosis events that have to be detected previously to centroid merging is defined by this setting.

Maximum frame border events

Similar to Maximum mitosis event. If the cell is detected several times at the frame border, the track terminates.

Delete cell at border

If this setting is selected, cells which migrated into or out of the field of view are deleted after the tracking process.

Initialise cells during processing

If this item is *not* selected, tracks are only initialized in the last frame (backward tracking) and at the frame border. If the item is selected, one can define up to which frame cell tracks are also initialized in the middle of the frame. As track initialization is always a crucial step in cell tracking, we would recommend to initialize up to the second or third frame and set an appropriate minimum track length[4].

Run two iterations

This is recommended. The tracking algorithm is run two times. In a first iteration the approximate speed of the cells is estimated and the *maximum centroid distance* is adjusted automatically. The second iteration runs then with more reasonable maximum cell displacements and is most likely to produce more reliable results.

Print info

Print out the progress of tracking in the command window.

Plot track image

The first frame of each processed video file is displayed and the extracted cell track are plotted into it. This can provide a quick glance at the data without the need to start the TLA result viewer 9.

[4]As we implemented backward tracking in this application, tracks *can* only emerge from the frame border during the analysis. Watching a cell video backwards means that cells do merge if a mitosis event occurs

8.3. Image batch-processing

This time lapse function provides the possibility of producing a single image from the video file. The image can either be a single extracted frame from the movie or a combination of up to all movie frames (e.g. "mean" frame). This time lapse analysis has no further settings yet. All settings must be adjusted on the image processing panel and the workstack. An example is provided on the TLA-webpage.

8.4. Movies

With the TLAit is possible to process movie frames iteratively and store the result in a new movie file. If the "movie" option is selected in the time-lapse pop-down menu the movie-panel will be shown. The *Video Quality* is a value between 0 and 100 and has no effect on uncompressed movie files. The higher the number, the higher is the video quality but the file size will also be larger.

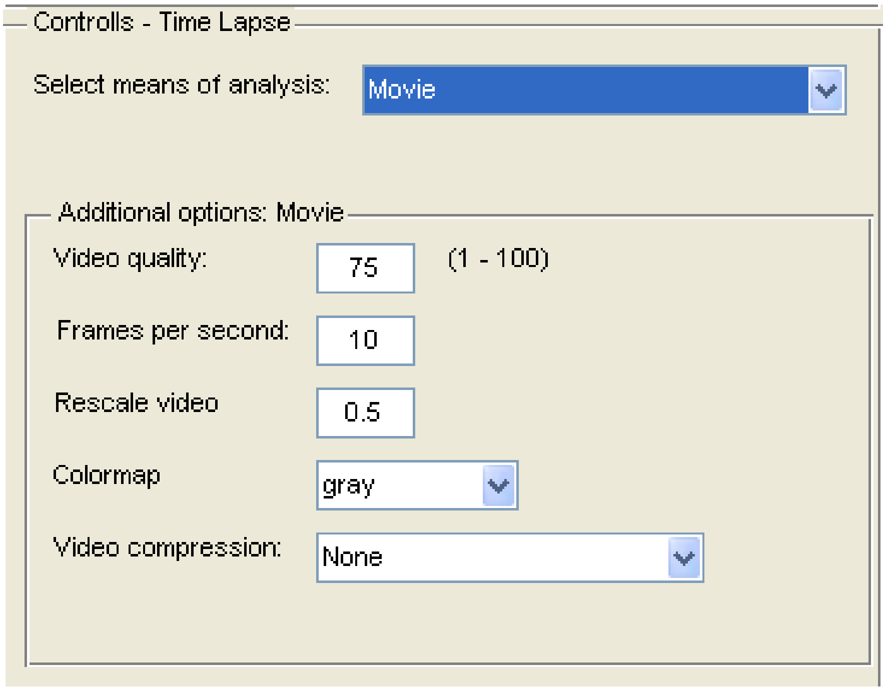

Figure 8.2:

Frames per second define the speed o the movie in frames that will be played in a second. The *Scale* option enables a scaling each frame before it is stored in the movie stack. The value *1* here denotes a scaling of 100%, thus does not influence the frame at all. The scaling can also be done in the workstack previously. Choosing a good colormap is done via the *Colormap* popdown menu. To test different colormaps and the effect on the movie frames see the Tools menu (5.4). The *Video compression* specifies the compression codec of the result video file. The compression option is only available on window operated systems in the current TLA version (v-0.1.2).

8.5. Alter and test time lapse settings

In some cases, the image processing and the time lapse analysis are independent. This is e.g. the case for the provided multi target cell tracking where the image processing has the sole purpose of

extracting cell centroids. For these cases, the possibility to test the time lapse analysis independent of the image processing is provided by the TLA.

The result files of a cell tracking experiment contain the cell tracks as well as the detected centroids of each frame. It is possible to load a result file, alter some tracking settings (MAP-item) and run the analysis again without running the time consuming image processing. The already extracted centroids will be reused with the altered tracking settings. Mind that if the time lapse settings are tested in this way, the results will not be saved automatically. To save a processed experiment use the menu item Save result as.

If a result file is opened and settings are altered *image processing*, the 'test time lapse' button will be disabled. This is due to the result file structure where all relevant informations are stored, which includes the image processing settings. If these stored settings are altered they will not "fit" to stored image processing results (e.g. centroids) which rises the need to process the images again.

Another useful function of the 'Test time lapse' button is to evaluate the time lapse settings on a list of already processed frames: if an analysis is started and then stopped again after e.g. 20 frames out of 100 are processed, one can already test the time lapse settings on these 20 frames. Again, the result is not stored automatically by the TLA.

9. Cell tracking result viewer

This section introduces the TLA-result viewer (RV) application for multi target cell tracking. The viewer is divided into two panels, the left contains at least the result file list (section 9.1), and the right panel provides graphical tools to analyze the tracking results (section 9.2). The left panel optionally holds video writing settings which are further explained in section 9.3. The RV menu entries are regarded in section 9.4.

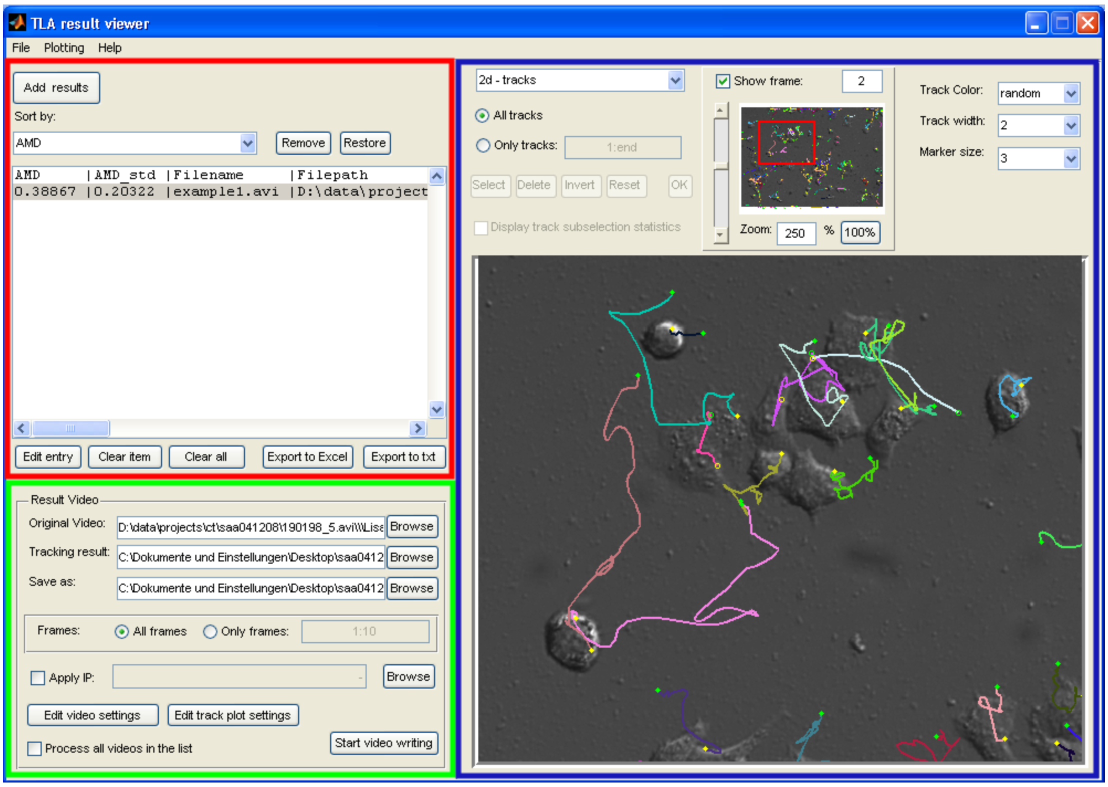

Figure 9.1: TLA-result viewer: overview of graphical interface and menu items. The RV consists of a result list section (red square), a video writing section (green square), and a graphical tool section (blue square).

9.1. Result list

Working with TLA results is simple. The results are presented in an expendable list with various provided functions to enable an effective data evaluation.

9.1.1. Working with entries

If the RV is invoked directly after an analysis by the TLA, all analysis results are automatically loaded into the result list. The 'Add results' button can be used to load results, which will then be included into the list.

Each list item consist of a fixed number of fields (e.g. Filename, AMD, AMD standard deviation, etc.). The entire list is always *sorted* regarding one of these fields (default is AMD). The sorting

can be customized using the pop-down menu directly below the 'Add results' button. The sorting is invoked with regard to the selected item.

If some fields are not necessary or even distracting they can be deleted. This is achieved by selecting the field name in the pop-down menu and hitting the 'delete' button right beside it. The 'reset' button restores all fields.

Below the list are buttons to modify list entries. One can edit a list entry, thus a tracking result by hitting the 'edit entry' button. Some fields of the actual selected entry can then modified. This function will be improved and more convenient in future versions of the RV. Entries can be deleted from the list, and the entire list can be cleared. Also, the export of the entire list into an MS-excel file format or into tab-spaced text files is supported.

9.2. Plotting results

The right side of the RV contains the graphical output of the time lapse results. The lower window shows a graphical response to the settings in the upper region. The possible adjustments include different types of plots, zoom functionality, track selection and marker selection possibilities.

9.2.1. Plot types

The different plotting types for TLA results are shortly introduced and particularities highlighted.

2d-tracks

All tracks are plotted into the window, the start of a track is marked by a yellow star, the track end by a green star. If a track terminated prematurely, or was initialized during the tracking process, the star markers are exchanged by circles, the color stays the same.

2d-tracks with track numbers

Same as 2d-tracks, only the track numbers are additionally plotted into the image.

Centroids

The computed centroids of the selected frame (see 9.2.4) are displayed. Sub-selection/deletion of centroids is not possible.

Equalize track origins plot

All tracks are translated so that their starting point lies at the point $(0, 0)$. This plot can be helpful for different purposes. First, the selection of exceptional "long" cell tracks is rather simple. Here, long refers to the fact that cells can cover a long distance, and the displacement between the first trajectory point and the last point is very high (for selection/deletion of tracks see 9.2.3). Secondly, using this display techniques directional gradients can be easily identified. The zoom function is not available for this plot type.

Mean displacement histogram

Plots a *mean displacement histogram* of the selected set of tracks. The track selection is restricted to 'invert' and 'reset', although single track numbers can also be selected/deleted from the track list (see 9.2.3). Zoom and frame selection is not available for this plot type.

MD box plots

Produces a box plot for each of the track sets in the result list (hence for each tracking result). This gives the possibility to compare the track sets with each other. Zoom and track selection/deletion is not available for this plot, as the entire result list is used rather than a single list entry. If one wants to delete exceptional fast or slow cells/tracks, up to now the possibility is to delete the tracks from the result file, store the result under a different name, and then reload it again.

9.2.2. Marker settings

On the upper right of the plot panel the track marker size, the track width and the color can be selected. Color selection is especially useful as the background of the plot image can change, i.e. due to plotting with or without background image. Yellow tracks are nicely visible on cell images, whereas they show only a poor contrast on the default white image background. In general the marker size defines the size of all point-like objects (e.g. the size of centroid markers in the centroid plot), and the track width defines the size of all line-like objects (e.g. the boxes of the box plots).

9.2.3. Result subsets

For some plotting types it is possible to select a subset of the cell tracks for the analysis. There are different means to achieve a subset selection. One possibility is to adjust the track list right beside the 'Only tracks' radio button. Here the *colon* operator can again be used (see section 8.1).

A more convenient way is often to select the tracks directly from the plot. Here, two different modes are provided: track selection and track deletion. In either case, if one hits the associated button the plotted tracks can be marked using the mouse pointer and left clicking on them. If selected tracks are clicked again, they are unselected.

To compare quickly between selected and not-selected tracks, the 'invert' button can be used. This will invert the selection, thus selecting previously selected/deleted cell tracks. The 'reset' function restores the entire track list.During track selection/deletion the track number list is constantly updated. By hitting the 'OK' button, the selection is applied and only the selected tracks are plotted.

9.2.4. Zoom and image display

The plot panel of the RV provides a zoom function of up to 800% which can often be useful if tracks are close together but should be selected individually. The zoom factor can be typed in or simply adjusted using the zoom slider left to the plot preview. In the plot preview, a red rectangle marks the region actual displayed in the main window. By clicking and dragging this rectangle, any region in the plot/image can be selected. The zoom function is not available for all plot types. If it is not available, it is simply not shown.

Note: In the current version of the RV (higher than v-0.1.2) the zoom switches back to 100% if the track subset selection function 9.2.3 is invoked. After the 'select' button was clicked, the zoom function can again be used.

9.2.5. Open separate figures

In the case, that individual displayed results should be presented in their own figure window, the function 'Open separate figures' can be used. A new window will be opened, displaying only the plot/image from the plot panel in the RV. The standard Matlab display window provides various functions to annotate and save the figure.

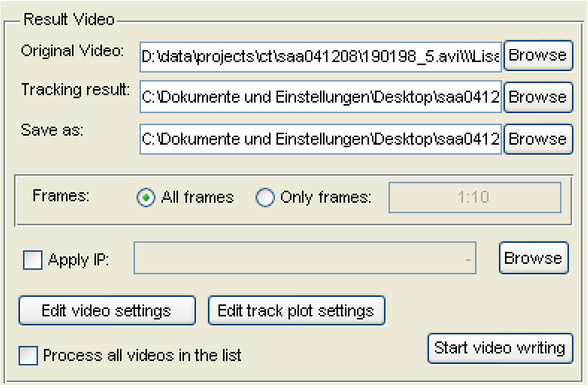

Figure 9.2:

9.3. Video options

If the cell video belonging to the actual viewed TLAresult file is located on the used computer, the resulting cell tracks can also be written directly into a result video file. The video file options can be switched on under menu entry Plotting→ Show video options

9.3.1. Files and directories

By clicking onto a result file in the result list (see 9.1), the video file path, the tracking result path and the result video file path are automatically adjusted. Changes can be made via the 'browse' buttons right beside the path fields.

9.3.2. Image processing options

The check box 'ApplyIP' can be used to apply image processing to each video frame previously to plotting the tracks into the video file, e.g. if the video contrast is extremely low. The RV uses TLA workstack routines. If image processing should be applied, the path to the appropriate workstack entry file must be selected via the 'browse' button. Informations as how to create workstack entries are in detail explained in section 7, how to save workstack entries is shown in section 7.7. Note that it is also possible to select entire setup files.

9.3.3. Track and video settings

In the lower section of the panel, two buttons are provided to adjust the video and the track plotting settings. Option dialogs will be opened, displayed in figure 9.3.

Video settings

The *quality* of the video can be adjusted as a value between 100% and 10%, where a higher number leads to a higher quality but also higher main storage needs. If the video options are tested on a machine with only a small amount of RAM available and huge frames are processed, the processing may be slow. The quality adjustments can help to reduce storage problems. Another way to avoid big files is to adjust the *Scaling* of the output file. Here values between 0.1 and 1 are allowed, where 1 will return the original size, and e.g. 0.5 rescale both the width and the height to half of their length.

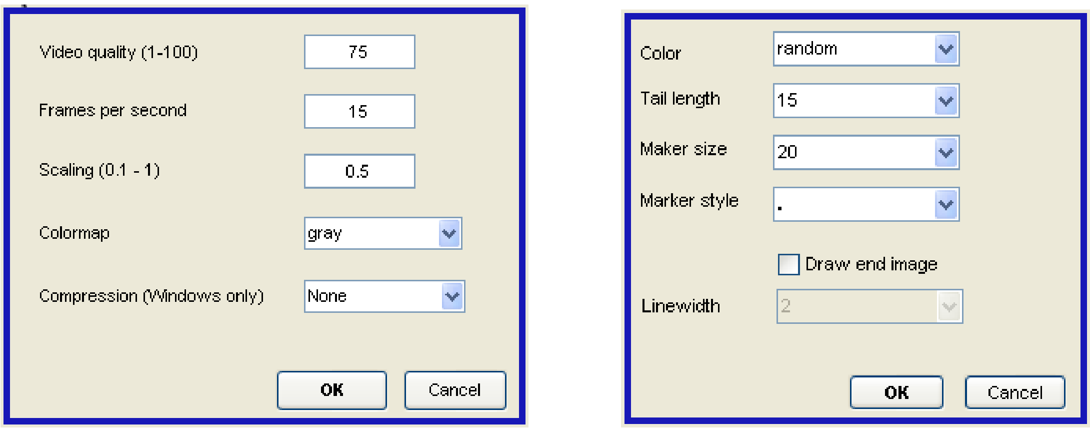

Figure 9.3:

We obviously do not have to explain *Frames per second* as it does pretty much what it looks like. The *Colormap* is another matter. The colormap will change the appearance of the images by pseudo-coloring them. The default map is "gray". The colormap option in the TLA can be used to test the appearence in advance.

The last setting, *Compression*, concerns only Windows operated systems (in the actual program version (v-0.1.2) of the RV). A Windows related compression format can be selected to significantly reduce the size of the resulting video files.

Track plot settings

Track plot settings concern the appearance of the cell tracks which are plotted onto the video frames. A cell track will be indicated by a certain marker with a special *Marker Style*, e.g. a dot, a rectangle or a circle. The symbol will mark the actual assumed position of the regarded cell. Per default, the tracks are plotted in random *colours* as to better identify one from another. The *Marker size* can also be selected which is especially needed if frames are strongly resized. The Marker size will not automatically adjust to the image size, so it has to be set here.

To provide better evaluation of cell speed, two features are provided: A *track tail* can be constructed by drawing also the markers of recent frames into the actual frame, only with slightly smaller markers. The effect reveals nicely the actual displacements of cells between frames. The number of recent displacements can be set under *Tail length*, up to 25 frames are possible. Another tool is the *line image* at the end which appends one additional frame at the end of the video file where all cell tracks are plotted as solid lines with an adjustable *line width*.

9.3.4. Batch processing files

If a set of results is loaded and for each result, a track video should be written, the box '☑Process all videos in the list' must be checked. The results will serially be processed with the actual video and track plot settings. The video files will automatically be written into the folders of the original video files.

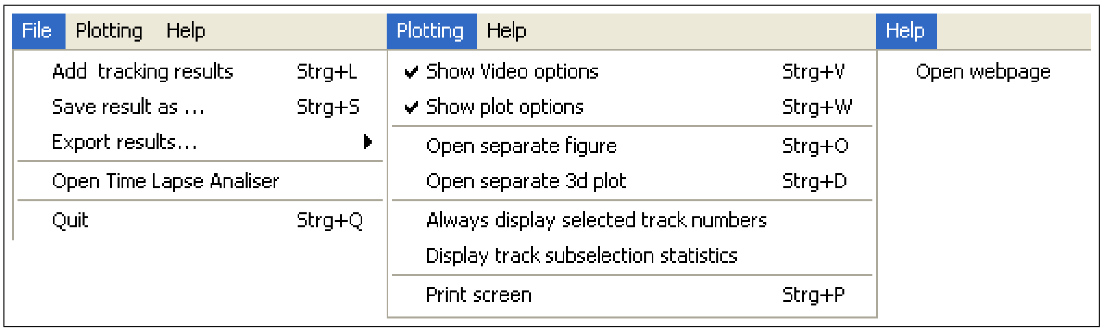

Figure 9.4:

9.4. Menus

9.4.1. File

- Add tracking results

  Works similar to the 'Add result' button. Appends TLA tracking results to the result list.

- Save results as

  Save actual highlighted item in the result list. While saving a result only the selected tracks are saved (see section 9.2.3). Also newly adjusted pixel sizes and image resolutions are saved in the result file.

- Export results

  Similar to the export buttons below the result list. Exports the result list either into text or excel format.

- Open Time Lapse Analyzer

  Opens the TLAif it is not already running.

- Quit

  Ends the program.

9.4.2. Plotting

- Show video options

  Toggles the video options panel below the result list panel on or off.

- Show plot options

Toggles the plot options panel right beside the result list panel on or off.

- Open separate figure

  Opens a separate figure window showing the actual displayed plot (See section 9.2.5).

- Open separate 3d plot

  Opens a separate 3d plot, displaying the tracks in the (x, y, t)-space. If the '☑Show frame' box is selected, the selected frame is also plotted into the figure. The figure provides zoom and rotation functions. These are rather slow if a frame is also displayed.

- Always display selected track numbers

  Displays the selected track numbers in the command window if any changes were made. This is a convenient function to validate long selected track lists.

- Display track subselection statistics

  If the AMD and the AMD standard deviation of the selected tracks should be displayed immediately after selection (hitting the 'OK' button), this menu entry has to be checked.

- Print screen

  Makes a screen shot of the RV.

9.4.3. Help

- Open webpage

  Opens the projects webpage. Here, updates and contact details of the authors can be found.

10. Interface for MatLab image processing functions

If the Advances options in the options menu are checked and the TLA was initialized from within a MatLab environment it is possible to include and test independent MatLab implemented image processing functions. The interface can be found on the image processing panel next to the quickoptions. One can adjust the image processing function file *function strImg = myip(strImg)* which has to have the following properties: strImg is a struct with fields *.org, .tmp, .pro*, where strImg.org is the orginal input image, *strImg.tmp* is a result from previous image processing operations (if any were applied), and *strImg.pro* has to hold the result file. The file myip.mat can be loaded clicking on the 'Edit' button. Another possibility is to select another function file, which needs to have the same input and output as myip().

The function will be called either previously to or after the workstack functions have been processed. One can also choose to test only the new code, which is similar to test it with an empty (only `'Original'`) workstack. In the current progress we are working on including arbitrary image processing functions directly into the workstack, which, apart from being more convenient, will be more powerful. Check the webpage (TLA homepage) for latest updates.

Note: The interface is not available if the TLA is used on a machine as stand alone application without a valid MatLab installation.

11. Further informations for MatLab developer

For those who like to include (or modify) image processing functions or new time lapse analysis directly in the code we will introduce the key concepts of our software in the next documentation update in the following sections. Until then, feel free to contact the authors for help.

A. WS-function listing

In general one has to be aware while using a certain unit with a basic operator. If used in combination with ('+,-,*,/'), a unit like 'MeanValue' returns the mean value of the *original* image. If combined with the 'usefor'-operator the mean value of the *so far processed* image is returned. More detailed information can be found in section 7, some examples are given both in section 7 and 7.6.

The next listing shortly explains the available WS functions. Most of the functions are explained more detailed and can be looked up in the Matlab™ online documentation.

If a function is not listed, it will hopefully be in the next documentation version. Feel free to contact the authors for help.

Some functions do not require additional settings (e.g. 'Adjust,Sharpen,Invert'), some functions need numerical input (e.g. 'Median(15),Entropy5'), and some functions need or accept numerical and character inputs (e.g. 'Scale(org),Scale(0.5)').

A.1. Image processing

- 'Original'
  Returns the original image. If used with 'usefor' it works as the identity function

- 'Median(mask)'
  Returns the (mask × mask) median filtered image

- 'Invert'
  Returns the inverted image. Inverted means that a gray value $g(x, y) \in [0\ 1]$ is replaced by $-(g(x, y) - 1)$. In general if $G_{\min}$ is the minimum gray value in the processed frame, and $G_{\max}$ is the maximum gray value, the invert operation returns $-(g(x, y) - G_{\max} - G_{\min})$

- 'Entropy(mask)'
  Computes the local image entropy at each pixel of the image in an $(mask \times mask)$ neighbourhood. The local image entropy is defined by $-\sum p * log(p)$ where p are the histogram counts of the neighbourhood.

- 'Blurring(mask)'
  Filtering with an $(mask \times mask)$ rectangle mean filter mask

- 'Log'
  Computes the natural logarithm on each image pixel of the image. As the pixel values normally range in between [0 1], this function must be handled with care

- 'MeanValue'
  Returns mean gray value of the image

- 'MedianValue'
  Returns median gray value of the image

- 'MinValue'

Returns minimum gray value of the image

- `'MaxValue'`
  Returns maximum gray value of the image

- `'Adjust(min,max,gamma)'`
  Increases the contrast of the image.

- `'Adjust(min,max,gamma)'`
  Increases the contrast of the image. The gray values are mapped into the interval by [`'min,max'`]. If `'gamma'` is one, the mapping is linear. If `'gamma'` is below 1, the mapping weights higher values stronger, and vice versa.

- `'Power(power)'`
  Replaces each gray value $g(x, y)$ in the image by $g(x, y)^{\text{power}}$

- `'Root(n)'`
  Replaces each gray value $g(x, y)$ in the image by the n'th root of $g(x, y)$

- `'Wiener(mask, noise)'`
  Wiener lowpass-filter in an $(mask \times mask)$ neighbourhood around each image pixel. For further information view. Wiener filter are used to denoise images.

- `'Bottom hat(threshold)'`
  Bottom hat transform. Suppresses local minima in the image. A gray value is defined a local minima, if its value lies `'threshold'` below the minimum gray value of its neighbourhood pixel. An (8×8) neighbourhood is taken.

- `'Top hat(threshold)'`
  Top hat transform. Suppresses local maxima in the image. A gray value is defined a local minima, if its value is at least `'threshold'` higher than the minimum gray value of its neighbourhood pixel. An (8×8) neighbourhood is taken.

- `'Erosion(mask)'`
  Erosion with an $(mask \times mask)$ filter mask with ones at every position.

- `'Dilation(mask)'`
  Dilation with an $(mask \times mask)$ filter mask with ones at every position.

- `'Opening(mask)'`
  Opening with an $(mask \times mask)$ filter mask with ones at every position. Equals `'(Erosion(mask),Dilation(mask))'`

- `'Closing(mask)'`
  Closing with an $(mask \times mask)$ filter mask with ones at every position. `'(Dilation(mask),Erosion(mask))'`

- `'Absolute Value'`
  Returns the absolute value of $g(x, y)$ at every image pixel

- 'Histogram Eq(gray value)'
  Performs a histogram equalisation with 'gray value' gray values.

- 'ScaleGrayValue(min,max)'
  Scales the gray values in the interval of ['min','max'].

- 'Gradient(size,direction,absolute)'
  Returns the x-gradient value of the image, where the gradient is computed as $\nabla x_n = (g(x,y) - g(x-n,y))/\text{size}$. If 'absolute' is 1, the absolute value of the gradient is returned which is equal to the WS-entry '(GradientX(n,0),Absolute Value)'

- 'Cutt off(threshold, setto)'
  Sets the value of the pixel with gray values below 'threshold' to the gray value 'setto'. 'threshold' can either be a numerical value, or 'mean' or 'median'.

- 'Delete between(threshold1,threshold2)'
  Sets the pixels with a value between 'threshold1' and 'threshold2' to zero.

- 'Set to maxthreshold'
  Sets the value of the pixel with gray values greater or equal to 'threshold' to the maximum occurring gray value in the image.

- 'ScaleImagescale'
  Rescales the image with the factor 'scale' in both the x- and the y-direction

- 'ScaleImagey,x'
  Rescales the image with the factor 'x' in x-direction and with factor 'y' in y-direction

- 'hmax(threshold,neighbourhood)'
  Suppresses regional maxima with a gray value which is not at least 'threshold' higher than the values of their surrounding neighbourhood. E.g. imagine a 1×6 image (line) with the following intensities: (3 3 10 3 7 3). The hmax transform with 'threshold' = 5 will return (3 3 5 3 3 3). One can see this operation as eroding all regional maxima up to either 'threshold' pixels, or until they are completely eroded and blend into their neighbouring pixels (see the value 7).

- 'hmin(thresh,neighbourhood)'
  Suppresses regional minima. Works similar to 'hmax'.

- 'LoG(mask,sigma)'
  Laplacian of Gaussian filter with size ($mask \times mask$) and standard deviation sigma.

- 'Disk(mask)'
  Disk shaped averaging filter mask within a squared box of side '2*mask+1'

- 'Motion(size,theta)'
  Returns an image which appears to be in movement in direction 'theta' with a step size of 'size'.

- 'Gaussian(mask,sigma)'
  Filters the image with a Gaussian lowpass filter of size ($mask \times mask$) and standard deviation 'sigma'

- 'Rangefilter(mask)'
  Returns the rangefilter image. each image pixel value is replaced by the range (maximum value-minimum value) of its ($mask \times mask$) neighbourhood. 'mask' has to be odd

- 'Standardfiltermask'
  Retuns the local standard deviation of an image in the ($mask \times mask$) neighbourhood.

- 'Standardfilter Edges(mask)'

- 'Sobelfilter Edges(threshold,direction)'
  Returns binary edge image. Sobel filter edge detection along direction 'direction'

- 'Prewittfilter Edges(threshold,direction)'
  Returns binary edge image. Prewitt filtered image with filtered edges whose value is higher than 'threshold'. Default direction is 'both', this can be changed to either "vertical" or "horizontal"

- 'Robertsfilter Edges(threshold)'
  Returns binary edge image. Roberts operator filtered image with filtered edges whose value is higher than 'threshold'.

- 'LoGFilter Edges(threshold,sigma)'
  Returns binary edge image. Laplacian of Gaussian filtered image with filtered edges whose value is higher than 'threshold'. The filter has the standard deviation 'sigma', the extend in x- and y-direction is automatically determined by ceil('sigma'*3)*2+1, where "ceil(.) is a Matlab function that rounds the argument to the next higher natural number."

- 'Cannyfilter Edges(T1,T2,sigma)'
  Returns binary edge image. Canny filter method of inding edges in an image. 'sigma' denotes the standard deviation of the Gaussian filtermask which is applied in advance. 'T1' defines the lower threshold, 'T2' defines the higher threshold for the canny edge detector. Edges start at values which are higher or equal to 'T2' and are followed as long as they do not fall beyond 'T1'.

- 'Next_n_Img(n,alternative)'
  While processing a video, one can refer to the next consecutive frame by this function. If the frame i is currently processed, this function returns image $i + n$. If this is not possible, the alternative image is returned which has the gray value 'alternative' at every image pixel. Default is a zero image.

- 'Previous_n_Img(n,alternative)'

While processing a video, one can refer to past frames by this function. If the frame i is currently processed, the image $i - n$ is returned. If this is not possible, the alternative image is returned which has the gray value `'alternative'` at every image pixel. Default is a zero image.

- `'Number of frames'`
  Returns the number of frames in the video file if all frames are selected or otherwise the number of selected frames.

- `'Actual frame number'`
  Returns the number of the frame that is actually processed.

- `'Rotate(degree)'`
  Rotates the image counter clockwise by `'degree'`-degrees. Note that up to now the binary rotated image cannot be merged with the original image. This will be improved in coming program versions.

A.2. Segmentation and binarisation

- `'Otsu'`
  Binarisation of the image using using the Otsu threshold as a global method

- `'Self defined(threshold)'`
  Global self defined threshold `'threshold'`

- `'Extended h-maxima(threshold)'`
  Local thresholding method.

- `'Extended h-minima(threshold)'`
  Local thresholding method.

A.3. Postprocessing

The next operations are all applied on already binarised images. Binarised images are divided into foreground pixels (value 1) and background pixels (value 0). Distinct clusters of foreground pixels in the images, like e.g. single cells or cell clusters are called regions. If every region has a single unique label, the image is called a label image.

- `'Fill in holes(maxregion)'`
  Fills in holes (black spots, zero regions) if the hole is smaller or equal than `'maxregion'`.

- `'Delete small regions(minregion)'`
  Deletes all regions which are smaller than `'minregion'`. This function can be used to eliminate background noise after binarisation.

- `'Delete large regions(maxregion)'`
  Deletes all regions which are greater or equal than `'maxregion'`.

- 'Erosion(mask size x, mask size y)'
  Applies morphological erosion on the image with a mask of size ('mask size x'×'mask size y'

. • 'Dilation(mask size x, mask size y)'
  Applies morphological dilation on the image with a mask of size ('mask size x'×'mask size y'

. • 'Opening(mask size x, mask size y)'
  Applies morphological opening on the image with a mask of size ('mask size x'×'mask size y'. This equals subsequent application of 'Erosion' and 'Dilation'.

- 'Closing(mask size x, mask size y)'
  Applies morphological closing on the image with a mask of size ('mask size x'×'mask size y'. This equals subsequent application of 'Dilation' and 'Erosion'

. • 'Remove interior pixels'
  Removes all interior pixels of a region. Only the border pixels of each region remain.

- 'Invert'
  Inverts the image. Image pixels with a former value one get the value zero, and vice versa.

- 'Centroidshift(mask,stepfac,method,minmax)'
  Shifts defined centroids (e.g. defined via the 'Centroids' command) toward extrema in the median filtered original image. The shift is gradient based. The highest (lowest) gradient in the area of ($mask \times mask$)around each centroid is computed and the centroid is shifted toward the regional maxima/minima; dependent on 'minmax'. If 'minmax' is 'max', regional maxima are found; if 'minmax' is 'min' minima in the image are found. The step size factor can be used to speed up the shifting process. 'Centroidshift' is normally only applied on region centroids. If the input image is not a centroid image, the regional geometrical centroids are at first computed.

- 'Centroids'
  Returns the regional centroids from the binary image. Input image must be binary.

B. Preparing setups for public use

There are some conventions which should be regarded while preparing TLA-setups for public use. In general, the publication should be packed (tar, zip) for download and including the following files:

Describe the script

Describe what the function/setup does and how it does it.

Include sample images

Make sure to include some frames from the tested movie files. This is necessary for TLA user to compare their own frame quality to the quality of the test frames. If videos with varying quality were used, make sure to include images from each.

Include information about possible error sources

If you can think of any possible errors, e.g. strongly varying image quality, image sizes, cell sizes etc. make sure these informations are included in the prepared setup.

Include processing times together with you machine specification and image sizes.

For many users speed is an essential parameter. Give a statement regarding the processing speed of the setup.

Include information about bottle necks

If there are any known bottlenecks in the analysis, (e.g. more than 100 cells will significantly reduce the power,...) name them in the publication.
